# High‐frequency sampling unveils biotic and abiotic drivers of rapid phytoplankton morphological changes

**DOI:** 10.1111/nph.70534

**Published:** 2025-09-04

**Authors:** Pavel Škaloud, Kateřina Tučková, Radka Čablová, Iva Jadrná, Ivana Černajová

**Affiliations:** ^1^ Department of Botany, Faculty of Science Charles University Benátská 2 12800 Praha 2 Czech Republic; ^2^ ENKI, o.p.s. – Třeboň Dukelská 145 37901 Třeboň Czech Republic

**Keywords:** community structure, DNA metabarcoding, environmental variables, freshwater ecosystems, high‐throughput sequencing, morphological traits, Synurales, zooplankton

## Abstract

- Phytoplankton, as primary producers, play a key role in aquatic ecosystems. Their community turnover is shaped by morphological traits that enable adaptation to diverse abiotic and biotic factors. Yet, the temporal scale of these dynamics remains poorly understood due to limited high‐frequency sampling studies.
- Employing DNA metabarcoding, we assessed the community composition of the phytoplankton lineage Synurales (Chrysophyceae) at 3‐d intervals during 70 d at a shallow peat bog lake in the Czech Republic. The selected group possesses a variety of species‐specific key morphological traits, such as cell size, coloniality, and bristle formation.
- Using a custom reference database of cultured species, we assigned 99.93% of eDNA reads to 74 species‐level lineages with known morphological traits. Community changes in colonial species were influenced by abiotic drivers such as silica concentration and wind speed. By contrast, shifts in unicellular species communities were mainly driven by Cladocera predators, influencing the occurrence of bristle‐bearing species.
- Changes in species composition and morphological traits occurred within days, mirroring environmental variability. Achieving such fine‐scale resolution, especially for small or rare taxa, would be extremely difficult using microscopy alone. eDNA enabled high‐resolution community profiling and abundance estimation, demonstrating its key role and the importance of comprehensive reference databases.

## Introduction

In the face of rapid and significant climate changes, understanding the temporal dynamics of biological communities has become increasingly important. Temporal changes refer to how communities of organisms evolve over time in response to various environmental factors. These changes are not constant; they fluctuate based on recent and historical events, influencing the structure and function of ecosystems. By studying these temporal patterns, ecologists can gain insights into the resilience and adaptability of species, predict future ecological shifts, and develop strategies to mitigate the impacts of climate change. This knowledge is crucial for preserving biodiversity and maintaining the health of our planet's ecosystems.

In freshwater aquatic ecosystems, phytoplankton as the primary producer plays a crucial role in the foundation of material circulation and energy flow (Harris, 1986). Temporal changes in freshwater phytoplankton communities are influenced by a variety of abiotic and biotic factors. Key abiotic factors include temperature, light availability, nutrient levels (especially phosphorus and nitrogen), and water mixing (Reynolds, 2006). Since these factors impact metabolism and growth, different phytoplankton species exhibit specific temperature preferences affecting their temporal dynamics (Marañón *et al*., 2013). In temperate regions, phytoplankton communities often undergo seasonal succession not only due to temperature shifts but also due to changes in light availability and nutrient content (Philips *et al*., 1997; Litchman, 2000; Shatwell *et al*., 2012). For example, chrysophytes prefer low temperatures, diatoms, and cryptophytes perform better with high nutrient supply, and green algae generally prevail under high light intensity (Salmaso, 2003; Marzetz *et al*., 2020). Phytoplankton composition can also be strongly affected by various abrupt weather changes, such as precipitation and storm events, which impact turbidity, transparency, and nutrient content (Reynolds, 2006; de Wit *et al*., 2016).

Biotic factors contributing to phytoplankton temporal dynamics include interspecific competition, interactions with other organisms, and the impact of grazing. Phytoplankton species often compete for limited nutrients (Litchman & Klausmeier, 2001) as well as for light in stratified waters (Philips *et al*., 1997). Some phytoplankton species engage in symbiotic relationships with other microorganisms, which can influence their temporal dynamics (Kazamia *et al*., 2016; Seymour *et al*., 2017). Several studies show that phytoplankton communities are strongly shaped by bacterial composition (Šimek *et al*., 2008, 2013; Bock *et al*., 2020). Finally, phytoplankton temporal changes are probably most affected by zooplankton and other grazers, who shift abundances of certain species and change phytoplankton composition (Reynolds, 2006; Lepori, 2019; Kovalenko *et al*., 2023).

Due to laborious microscopic analyses, the vast majority of temporal studies conducted on freshwater phytoplankton were generally based on monthly, or less commonly, weekly sampling (Reynolds, 1980; Siver & Chock, 1986; Siver & Hamer, 1992; Padisák *et al*., 1998, 2010; Willén, 2003). However, such low sampling frequencies are inadequate for capturing the rapid dynamics of protists, which have short generation times and can respond very quickly to environmental changes (Lehman & Sandgren, 1985; Payne, 2013). This limitation was overcome by the introduction of high‐throughput sequencing (HTS) and the availability of DNA metabarcoding techniques, also called amplicon metagenomics or amplicon sequencing, enabling studies based on large diversity datasets and shorter sampling frequencies (Simon *et al*., 2015; Mukherjee *et al*., 2017, 2024). On the other hand, DNA metabarcoding results are strongly affected by the quality of reference taxonomic databases, which often lack genetic information for many species, hindering accurate species identification of several identified taxa. Accordingly, modern studies generally focus on the abundance of higher taxonomic levels, such as classes or phyla (Rolland *et al*., 2009; Alvarez‐Cobelas *et al*., 2019; Bai *et al*., 2023; Mukherjee *et al*., 2024), with a limited understanding of the seasonal dynamics of individual species. This results in the loss of valuable information on species‐specific morphological traits, which are crucial for ecological studies, as they can provide insights into species' adaptations, interactions, and roles within their ecosystems.

To address these challenges, we utilized DNA metabarcoding with high‐frequency sampling every 3 d, specifically targeting the order Synurales (Chrysophyceae). By generating a custom‐curated reference taxonomic database for species within this group, we achieved precise species‐level identification of the observed genetic diversity. This approach allowed us to overcome the limitations of both traditional microscopic analyses and modern HTS, providing a more accurate and detailed understanding of phytoplankton diversity and dynamics.

**Fig. 1:**
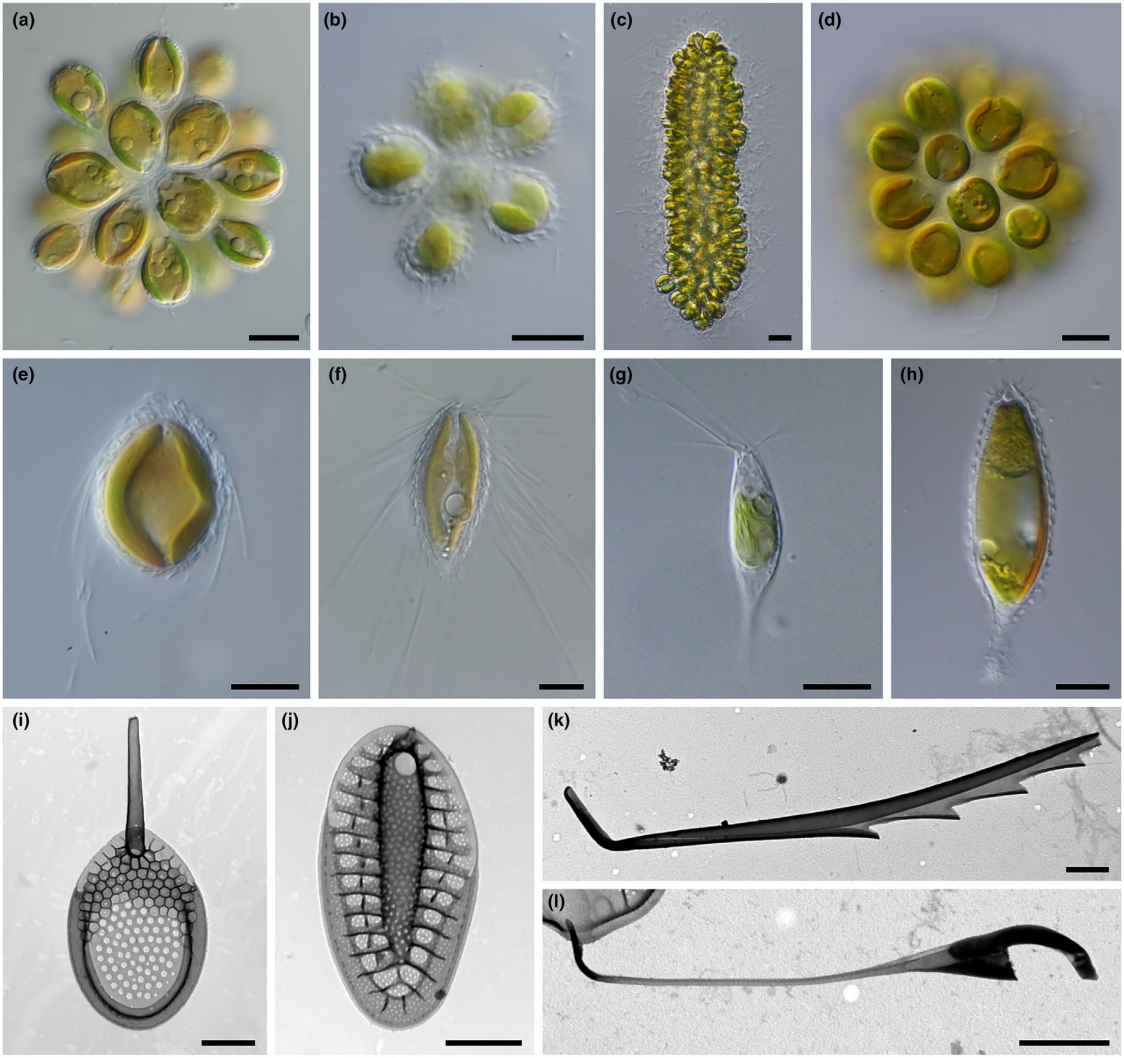
Morphological traits of the model lineage Synurales. (a–d) Different types of coloniality. (a) Smooth colony with elongated cells (*Synura petersenii*). (b) Spiny colony (*S. echinulata*). (c) Elongated colony (*S. punctulosa*). (d) Spherical colony with spherical cells (*S. glabra*). (e–h) Bristle arrangements in unicellular species (LM). (e) Bristles formed in the anterior 2/3 of the cell (*Mallomonas intermedia*). (f) Long bristles formed uniformly across the cell surface (*M. caudata*). (g) Bristles formed just at the anterior cell end (*M. akrokomos*). (h) Bristles absent (*M. insignis*). (i, j) Detailed structure of silica structures (transmission electron microscopy; TEM). (i) Scale with spine (*S. spinosa*). (j) Scale with central keel (*S. petersenii*). (k) Serrated bristle (*M. caudata*). (l) Helmet bristle (*M. heterospina*). Bars, 10 μm (a–h), 1 μm (i–l). LM, light microscopy.

The order Synurales is found in various freshwater habitats, contributing to primary production and serving as a food source for various microorganisms and small invertebrates (Kristiansen & Škaloud, 2017). The order is distinctive in that the species included are not only known to be sensitive to abiotic factors, primarily pH and conductivity (Siver, 1995; Jadrná & Škaloud, 2025), but they have also developed various strategies to cope with predation, such as adopting a colonial lifestyle and producing silica scales and bristles with unique morphology (Kristiansen & Preisig, 2007). Synurales includes two major genera of flagellates, colonial *Synura* (Fig. 1a–d) and unicellular *Mallomonas* (Fig. 1e–h). Two scale types can be recognized in *Synura*. The first type, found in sections Curtispinae and Synura, has flat scales with a spine (Fig. 1i). Colonies of this scale type are spiny due to the presence of spines protruding out of the colony (Fig. 1b). The second type, found in section Petersenianae, lacks a spine but features a prominent keel running along the center of the scale (Fig. 1j; Škaloud *et al*., 2020). Colonies of this scale type appear much smoother (Fig. 1a,d). Several species with spiny colonies are also known to form elongated or filiform colonies (Fig. 1c), whereas Petersenianae forms only spherical colonies (Fig. 1d). The bristles are formed only by *Mallomonas* species and vary in length and shape (Fig. 1k,l). They may be distributed uniformly across the cell surface (Fig. 1f), formed only at one or both ends of cells (Fig. 1g), or they can be completely absent (Fig. 1h) (Siver, 1991).

Using Synurales as a model group, this research aims to capture rapid community changes and trace how abiotic and biotic factors influence community structure. Specifically, we seek to uncover the temporal dynamics of phytoplankton community composition by addressing several key questions: (1) What are the time scales over which changes in phytoplankton communities occur? (2) Which abiotic and biotic factors are directly related to these community changes? (3) How are specific morphological traits within Synurales – such as cell size, coloniality, scale type, and the presence of spines – influenced by the studied abiotic (physical–chemical parameters, climate) and biotic factors (zooplankton community composition and functional traits)? By addressing these questions, the study hopes to enhance our understanding of the mechanisms driving phytoplankton diversity and distribution in freshwater ecosystems.

## Materials and Methods

### Study area and sampling design

Sampling was performed at the peat bog lake Spálená Borkovna, located near the town of Třeboň, Czech Republic (48.9800389N, 14.7994933E). It is an artificially created water body due to sulphur–iron peat extraction, which began in 1960 and continues until now. The bog is *c*. 3.5 ha in size, with a shallow littoral, a muddy bottom, and a gradual bank at the sampling site. The site was visited every 3 d at noon from 7 April to 14 June 2021, resulting in a total of 24 samples. At each sampling event, we collected: a phytoplankton sample using a sterile plankton net with a mesh size of 20 μm, a zooplankton sample using a sterile plankton net with a mesh size of 100 μm – *c*. 700 l were filtered in total across both net samples, and a 200 ml water sample for chemical analyses. The plankton nets were sterilized before use with a 4% bleach solution. We also measured water temperature, pH, and conductivity using a WTW 340i (WTW GmbH, Weilheim, Germany). After each sampling, the phytoplankton sample was mixed thoroughly, a small portion was fixed for storage by adding a formaldehyde solution (final concentration 10%), and the rest was frozen at −20°C for DNA metabarcoding analyses (see ‘DNA metabarcoding, bioinformatics’ in the Materials and Methods section). The zooplankton sample was fixed by adding a formaldehyde solution (final concentration 4%), and the water sample was processed as described below.

### Abiotic data

Abiotic data consisted of measured values of temperature (°C), conductivity (μS cm^−1^), and pH (as mentioned in the previous section), concentrations of selected nutrients, and climatic data (Supporting Information Table S1). The total nitrogen (TN, mg l^−1^), total phosphorus (TP, mg l^−1^), and total carbon (TC, mg l^−1^) contents were obtained by analyzing sampled water fixed by H_2_SO_4_ (TN), HNO_3_ (TP), using spectrophotometry following ISO 11905‐1 (TN), ICP‐MS following ISO 17294 (TP), and thermal oxidation following ISO 1484 (TC). Concentrations of silica (Si, mg l^−1^) and calcium (Ca, mg l^−1^) were measured by ICP‐OES following ISO 11885 using sampled water filtered through 0.45 μm Polyethersulfone syringe filters and fixed by HNO_3_. Chemical analyses were performed in Aquatest (Prague, Czech Republic). Measurement uncertainties represent expanded uncertainties calculated with a coverage factor of *k* = 2, corresponding to a 95% confidence level. Specifically, the uncertainty for TP measurements was ±25%, for TC ±15%, and for Ca ±15%. Climatic data were obtained from the Czech Hydrometeorological Institute, based on measurements at weather station C2TREB01 located 9.5 km from the sampling site (49.0622222N, 14.7586111E). Climatic data included average wind speed (m s^−1^), average daily relative humidity (%), daily precipitation (mm), daily sunshine duration (h), average daily air temperature (°C), maximum daily air temperature (°C), and minimum daily air temperature (°C). Since plankton dynamics can also be influenced by past climatic changes, values of precipitation and wind speed were obtained not only for the sampling day but also for the previous day.

### Biotic data

The zooplankton samples were used to assess the diversity of the main Synurales grazers, that is Copepoda, Cladocera, and Rotifera (Table S2). Each sample was washed in distilled water, homogenized, and then 0.3 ml of the sample was pipetted onto a 26 × 76 mm slide and covered with a 24 × 50 mm coverslip. Five to 10 microscopic slides were processed under a Carl Zeiss Axioskop light microscope and at least 50 randomly selected adults were identified to the species level. To account for the variable number of determined zooplankton species, each sample was normalized to 100 individuals for subsequent statistical analyses.

### ITS rDNA reference database of Synurales

In order to identify Synurales species present in DNA metabarcoding data, we created a custom internal transcribed spacer (ITS) rDNA reference database based on extensive isolation and morphological determination of strains during 2007–2023 (Table S3). The strains were isolated from various European localities by micropipetting individual cells or colonies into 96‐well plates and cultivated as described in Škaloud *et al*. (2020). Species identification was based on transmission electron microscopy (TEM) examinations of silica scales. ITS rDNA barcode sequences were obtained by Sanger sequencing as described in Jadrná *et al*. (2021), using the primers described there or the primers Kn1.1 (5′‐CAA GGT TTC CGT AGG TGA ACC‐3′; Wee *et al*., 2001) and ITS4 (5′‐TCC TCC GCT TAT TGA TAT GC‐3′; White *et al*., 1990).

### DNA metabarcoding, bioinformatics

Environmental DNA (eDNA) extractions were performed using the DNeasy PowerWater Kit (QUIAGEN), applied to 2 ml of thawed and sedimented material pipetted from the bottom of the plankton net sample container. ITS1 rDNA amplicons were produced by PCR using the newly designed primers Chryso‐SSU1 (5′‐GGT GAA GTC GTA ACA AGG TTT CCG TAG G‐3′) and Chryso‐5.8S5 (5′‐CAG AYA CTC CAR CAG ACA TRC‐3′), preferentially amplifying Chrysophyte organisms. To these primers, 8‐base sample‐specific barcodes were attached to differentiate among the samples. Moreover, three fully degenerated positions (Ns) were added to the primer 5′ end to enhance molecular diversity during sequencing. All PCRs were prepared in a 20 μl volume consisting of 10 μl Q5 Master Mix (Q5 High‐Fidelity DNA polymerase, BioLabs Inc.), 1 μl of each forward and reverse primer, 30–40 ng of DNA template, and water. The PCR profile included initial denaturation at 98°C for 30 s, 35 cycles of denaturation at 98°C for 10 s, amplification at 52°C for 45 s and elongation at 72°C for 1 min, and a final extension at 72°C for 2 min. Each sample was run in two replicates. If one of these replicates produced a low number of reads, a third replicate was conducted and the resulting reads were merged with those from the low‐reads replicate to ensure sufficient data quality and quantity. We further included 20 PCR negative controls (with distilled water as template) and 20 multiplexing controls (unused combinations of left and right barcodes). The PCR products were purified with SPRI AMPure XP paramagnetic beads (Beckman Coulter) and pooled equimolarly. HTS library preparation and Illumina MiSeq paired‐end sequencing (2 × 300 bp) were performed at Fasteris (Genesupport SA, Plan‐les‐Ouates, Switzerland). Libraries were prepared using the Metafast Metagenomics v.2 protocol, a Fasteris in‐house PCR‐free workflow. This involved 3′ adenylation and ligation of indexed adapters, without a final PCR amplification step, helping to reduce PCR‐induced chimera formation. Two HTS libraries were prepared, each containing one PCR replicate of every sample and negative controls.

Quality control of the Illumina MiSeq paired‐end reads was carried out using FastQC v. 0.11.8 (Andrews, 2010). Raw reads were processed following the protocol described in Bálint *et al*. (2014), using scripts provided in that publication's supplementary material. This included quality filtering (Perl script), paired‐end assembly (PANDAseq; Masella *et al*., 2012), removing primer artifacts (Python script), reorienting reads to 5′‐3′ (fqgrep; https://github.com/indraniel/fqgrep), demultiplexing by barcode combinations (fqgrep), removing primers with barcodes (Fastx Toolkit; http://hannonlab.cshl.edu/fastx_toolkit/), and dereplication with generation of unique hash values (vsearch; Rognes *et al*., 2016). Clustering was performed by Swarm v.2 (Mahé *et al*., 2015) with the clustering threshold set to *d* = 3, followed by chimera filtering (vsearch), generating a total of 72 768 denoised amplicons (swarms). To avoid spurious sequences originating from PCR errors, only swarms that were found in at least three reads and in both replicates were considered. The swarms were identified by Blast searches in SEED2 (Větrovský *et al*., 2018), using both remote and local Blast searches. The local ITS rDNA reference database was created using the makeblastdb command (NCBI, MD, USA), based on the Synurales sequences obtained as described above. Only Synurales sequences were further processed, that is, a total of 1862 swarms. Of these, nine were present in negative controls of one of the replicates, so the resulting abundances were considered only from the other replicate. For the other swarms, the higher abundance from both replicates was selected for each sample. Finally, all swarms were aligned using Mafft v.7 under the Q‐INS‐I strategy (Katoh *et al*., 2019) and a maximum likelihood phylogenetic tree was constructed in iq‐tree v.1.6.1. (Nguyen *et al*., 2015) using GTR + I + G substitution model. The tree topology was tested using ultrafast bootstrapping with 1000 replications implemented within the same software. All swarms were then visually inspected to identify chimeric sequences and pseudogenes, and this selection was compared with the one carried out by vsearch chimera filtering. Visual inspection greatly corroborated automatic chimera detection with the exception of four swarms where vchime did not identify chimeric sequences, and one swarm identical to the *Mallomonas* Perty reference sequence wrongly identified as a chimera. After removing chimeric sequences and pseudogenes, the final phylogenetic tree was inferred by RAxML 8.2.12 (Stamatakis, 2014) to phylogenetically define the species. The analysis was performed under the GTR + I + G substitution model, with the rapid bootstrapping procedure using two independent runs and 1000 pseudoreplicates. Finally, all swarms belonging to the same phylogenetically defined species were merged and their abundance was summed (Table S4). To account for variable number of reads, each sample was normalized to the 15^th^ percentile of the most abundant sample (43 894 reads) using the ‘rarefy_even_depth’ function in the phyloseq package (McMurdie & Holmes, 2013) in R (R Core Team, 2023). Four samples with lower abundance were normalized to 43 894.

### Functional traits

After identification, each *Mallomonas*, *Synura* Ehrenberg, and zooplankton species was characterized by measuring or determining several functional traits (Tables S2, S4). Our reference strain database was used to obtain morphological traits for genetically identified species using DNA metabarcoding. In *Mallomonas*, we measured average cell length, cell width, and bristle length based on six randomly selected cells and estimated the cell volume using the formula for the volume of an ellipsoid. Additional functional traits included the ability to form bristles and the pattern of bristle formation, specifically analyzing whether the bristles are formed all around the cell or only partially. In *Synura*, we measured average cell length, cell width, and colony dimensions based on six randomly selected colonies and estimated both the cell volume and the colony volume using the formula for the volume of a sphere or cylinder. Additional functional traits included colony shape and production of spiny colonies due to the formation of apical spines on silica scales.

For zooplankton species, these traits included higher taxonomic rank (Cladocera, Copepoda, Bdelloidea, and Monogononta), maximum body size, trophic group (nonpredators and predators), feeding type (capture, verification, primary filtration, secondary filtration, suction, and gathering), and locomotion (swimming, crawling, attachment). With the exception of taxonomic rank, all functional traits were obtained from Gavrilko *et al*. (2020).

Finally, the functional traits described above were used to characterize temporal patterns and changes in the community by summing the abundances of species with specific traits or by estimating the average trait values for each collection date (Table S5).

### Statistical analyses

Ordination analyses were conducted using Nonmetric Multidimensional Scaling (NMDS) in the vegan package in R (Oksanen *et al*., 2022; R Core Team, 2023), employing the Bray–Curtis dissimilarity index to assess community composition. To explore the relationships between NMDS scores and environmental factors or zooplankton traits, Pearson correlation analyses were performed. These correlations were visualized using the ordisurf() function in the vegan package. Temporal changes were visualized in R, using the ggplot2 and ggalluvial packages (Wickham, 2016; Brunson, 2020).

To test for the significant effect of environmental variables and zooplankton functional traits on Synurales temporal patterns, we performed the model selection using the mumin package in R (Kamil, 2016). First, we reduced the number of predictors by performing a correlation analysis using Pearson's correlation coefficients to identify significantly correlated variables by hierarchical clustering using the corrplot package in R (Wei & Simko, 2024). As a result, a total of seven environmental variables (TC, TP, silica concentration, pH, daily precipitation, average wind speed, average air temperature) and 8 zooplankton traits (% of Cladocera, % of Copepoda, % of Bdelloidea, % of Monogononta, feeding by capture, feeding by primary filtration, feedig by secondary filtration) were selected. With the set of 15 selected variables, we fitted a full model with the whole set of predictors for each phenotypic trait and then performed an all‐subsets model selection based on AIC_c_ (Second‐order Akaike Information Criterion), comparing models with all combinations of predictors using the dredge() function. We then summarized the outputs of these models using the strategy published by Mašková *et al*. (2022). First, we subsampled all models that differed from the best model by < 10 Akaike units. Then, the relative importance of each predictor was estimated by the sum of the AIC_c_ weights of the models in which the given predictor appeared, divided by the sum of the AIC_c_ weights of all models. Finally, for each predictor, the standardized regression coefficients and their confidence intervals were determined by computing their Akaike‐weighted means (exp(−0.5) * deltaAIC_c_ values) from all models that included that term. The regression coefficient plots were generated using the ggplot2 package in R. For traits where the top model outputted high *R* ^2^ values, trait‐environment relationships were visualized using regression plots. Initially, trait‐predictor relationships were modeled using linear regression (lm function in R), and we performed diagnostic tests to assess standard assumptions of linear modeling. These included visual inspection of residual vs fitted plots for linearity and homoscedasticity, Shapiro–Wilk tests for residual normality, and evaluation of Cook's distance to identify influential data points. Where assumption violations were evident – particularly in relationships showing V‐shaped residual patterns or poor fit – generalized additive models (GAMs) were applied using the mgcv package in R to account for potential nonlinearity. These GAMs improved explanatory power (*R* ^2^) and more accurately represented biological trends.

## Results

### Abiotic and biotic explanatory factors

During the 70 d of the investigation, we observed fluctuations in daily precipitation and sunshine duration with notable peaks and troughs (Table S1, Fig. S1). Both water and air temperatures exhibited a gradual increase over the study period, reflecting the seasonal transition from spring to early summer. While TC concentration increased during the study period, TP levels decreased, with the exception of a single peak on the 46^th^ d of the investigation. TN and Ca concentrations exhibited similar trends, both showing distinct increases just after the last precipitation event on the 37^th^ d. Other measured parameters showed slight variability but did not exhibit clear increasing or decreasing trends. The pH levels of the water remained fairly constant, indicating stable acidity levels.

The zooplankton community exhibited remarkable diversity, with a total of 47 species identified, indicating a very rich community (Table S2). Cladocera were the most abundant group, comprising *c*. 64% of all individuals. The most abundant species within this group were *Chydorus sphaericus*, which accounted for more than half of the occurrences, and *Alonella nana*. Rotifera were the second most abundant group, making up *c*. 27% of the total. The vast majority of Rotifera belonged to Monogononta, with *Kellicottia longispina* being the most prevalent species. Finally, Copepoda were quite rare, constituting < 4% of the total abundance.

### Synurales reference database

A total of 106 strains representing separate species‐level evolutionary lineages were included in the ITS rDNA reference database, including strains morphologically belonging to 73 *Mallomonas* and 20 *Synura* described taxa (Table S3, Fig. S2). Accordingly, this resulted in the successful acquisition of 63% of *Mallomonas* and 69% of *Synura* taxa ever recorded in Europe (Škaloud *et al*., 2013), encompassing all commonly found species. Notably, in many cases (e.g. in *M. akrokomos*, *M. striata*, *M. papillosa*, *M. tonsurata*), we identified the presence of several cryptic taxa, which are genetically distinct yet morphologically classified under a single taxon.

### Species diversity and temporal patterns

A total of 74 species and species‐level Synurales lineages were recovered during the temporal study (Table S4). Only four lineages did not match any of the strains included in the reference database, meaning 95% of all lineages matched morphologically characterized reference strains (Figs S3, S4). When considering the abundance of reads, 99.93% of all reads could be attributed to morphologically defined strains included in the reference database. The unicellular species were much more diverse than the colonial species, with 81% species or species‐level lineages retrieved. However, colonial species were markedly more abundant, with 2 653 098 reads compared to 76 222 reads for unicellular ones. This indicates that 97% of the biomass was accounted for by colonial species, given their cell sizes are more or less comparable to those of unicellular taxa. The most abundant species were *Synura glabra*, *S. macropora*, and *S. spinosa*, which together constituted 76% of all Synurales reads.

The temporal patterns revealed by eDNA metabarcoding highlight the method's high sensitivity and resolution in capturing rapid changes in species diversity, including those occurring at short time scales and under low‐density conditions. The nearly complete assignment of sequence reads to morphologically characterized species allowed for accurate reconstruction of community dynamics and fine‐scale trait variations.

**Fig. 2:**
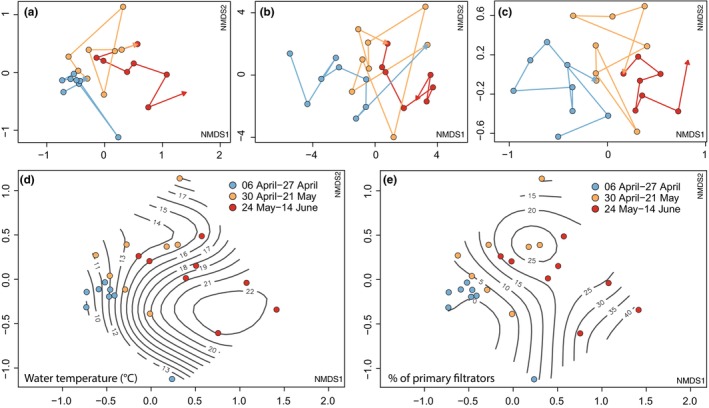
Temporal dynamics and environmental associations in the Synurales community. Temporal trajectories of the Synurales community (a), environmental parameters (b), and the zooplankton community (c) in the peat bog lake Spálená Borkovna, identified by nonmetric multidimensional scaling (NMDS) analyses. Points are connected by arrows indicating sampling progression over time, with colors representing three time periods as shown in the legend within the figure. (d, e) Contour plots show the relationships between NMDS scores of the Synurales community and water temperature (d) and the proportion of primary filtrators (e).

The NMDS analysis revealed a clear temporal shift in community composition, with early samples clustering distinctly from those collected later in the study period (Fig. 2a). A similar temporal pattern was observed in both environmental parameters (Fig. 2b) and zooplankton communities (Fig. 2c). For environmental data, the first NMDS axis showed strong correlations with TC (Pearson correlation = 0.77) and water temperature (Pearson correlation = 0.73) (Fig. 2d). In the case of zooplankton, the first NMDS axis was most strongly correlated with locomotion by attachment (Pearson correlation = 0.77) and feeding by primary filtration (Pearson correlation = 0.64) (Fig. 2e). Accordingly, time played a crucial role in shaping the community dynamics of both Synurales algae and their predators.

**Fig. 3:**
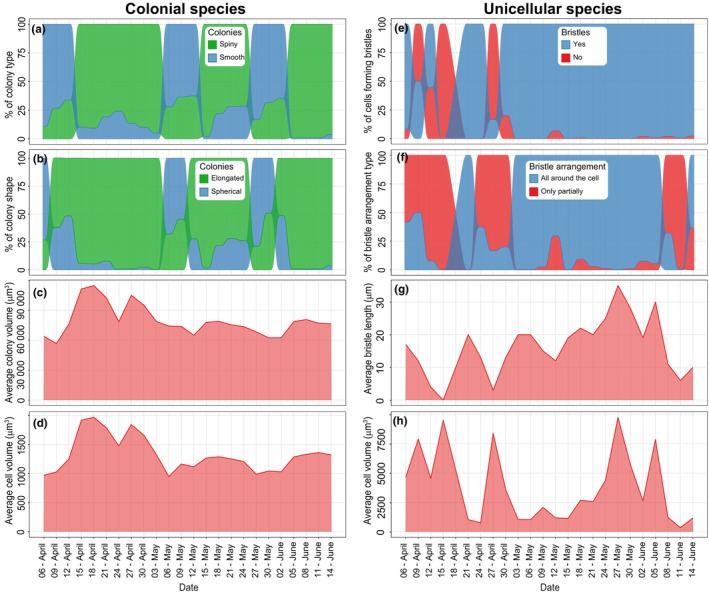
Temporal plots of morphological trait changes in colonial (a–d) and unicellular (e–h) species. (a) Spiny and smooth colonies, (b) elongated and spherical colonies, (c) average colony volume, (d) average cell volume, (e) proportion of cells forming bristles, (f) proportion of bristle arrangement type, (g) average bristle length. (h) Average cell volume.

In colonial species, we observed distinct cyclical changes in colony characteristics, with transitions between spiny and smooth types (Fig. 3a) and shifts from elongated to spherical shapes (Fig. 3b) occurring *c*. every 4–7 d. Average cell and colony volumes (Fig. 3c,d) were highly correlated and were notably larger in the early samples and decreased in size over time. Unicellular species exhibited notable changes in bristle formation (Fig. 3e) and arrangement (Fig. 3f), though these changes were not cyclic and were predominantly observed in the early samples. The average cell volume (Fig. 3g) and bristle length (Fig. 3h) also showed distinct variations, with these traits being inversely related in the early samples but aligning more closely in the later ones.

### Effects of abiotic and biotic variables on temporal morphological patterns

**Fig. 4:**
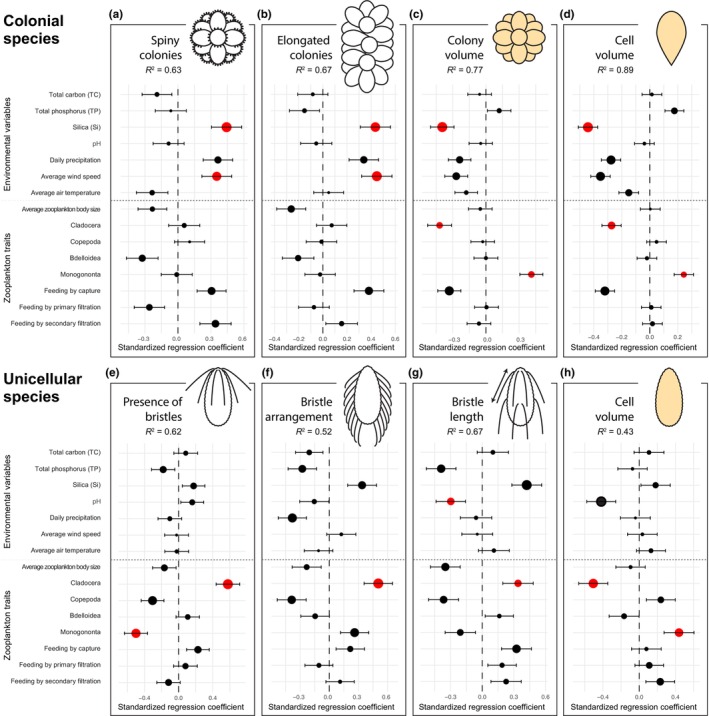
Effects of environmental variables and zooplankton traits on morphological traits in colonial (a–d) and unicellular (e–h) species. Traits analyzed are spiny colonies (a), elongated colonies (b), average colony volume (c), average cell volume within a colony (d), presence of bristles (e), bristle arrangement (f), bristle length (g), and average cell volume (h). Points represent estimation of standardized regression coefficients, whereas the error bars correspond to their 95% confidence intervals, calculated from the distribution of coefficients across all models in which the predictor appeared. Point size is proportional to the relative importance of the given predictor (i.e. the weighted occurrence of the predictor in the set of best models); predictors with significant net effect (*P* < 0.05) are given in red. Shaded cells and colonies indicate traits used in volume regression.

A number of environmental variables and zooplankton functional traits significantly affected the temporal dynamics of morphological traits (Fig. 4).

**Fig. 5:**
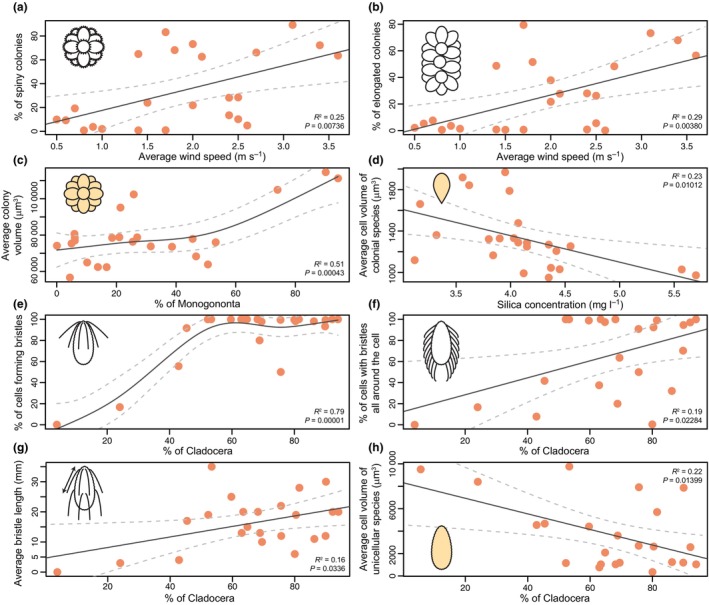
Regression models showing the most significant relationship between morphological traits and ecological variables in colonial (a–d) and unicellular (e–h) species. Traits are associated with average wind speed (a, b), Monogononta relative abundance (c), silica concentration (d), and Cladocera relative abundance (e–h). Linear models (LM) were used unless specified; plots (c) and (e) were fitted using generalized additive models (GAMs) to better capture nonlinear trends. Significance values (*P*) were derived from *F* tests for LMs and from smooth term significance in GAMs. Shaded cells and colonies indicate traits used in volume regression. Dashed lines represent 95% confidence intervals around the fitted regression lines.

In colonial species, the type and shape of colonies were influenced by environmental variables, primarily by silica concentration and average wind speed (Fig. 4a,b). Both spiny and elongated colonies were significantly more prevalent in windy conditions (Fig. 5a) and with high silica concentrations (Fig. 5b). Colony and cell volumes were affected by both abiotic (silica concentration) and biotic factors (the relative abundance of Cladocera and Monogononta predators) (Fig. 4c,d). The correlations were consistent for both cell and colony volumes, with higher volumes observed under increased relative abundance of Monogononta (Fig. 5c) and lower silica concentrations (Fig. 5d).

In unicellular species, bristle formation and arrangement were significantly influenced by zooplankton traits (Fig. 4e,f). The relative abundances of Cladocera predators played a crucial role; when Cladocera made up more than 50% of the zooplankton community, a majority of species were bristle‐bearing (Fig. 5e). Similarly, as the proportion of Cladocera increased, there was a higher abundance of species with bristles covering the entire cell surface (Fig. 5f). The presence of Cladocera also affected bristle length, with higher Cladocera percentages correlating to a fourfold increase in average bristle length (Fig. 5g). The bristle length was also influenced by pH values, with longer bristles being formed under lower pH conditions (Fig. 4g). Finally, the cell volume of unicellular species was significantly correlated with the relative abundance of Cladocera (Fig. 4h). While a high relative abundance of Cladocera stimulated bristle production (as mentioned in the previous section), it was correlated with a decrease in the average cell volume (Fig. 5h). Communities composed of larger cells were, on the other hand, associated with a higher relative abundance of Monogononta predators (Fig. 4h).

## Discussion

### Rapid morphological changes in a phytoplankton community

**Fig. 6:**
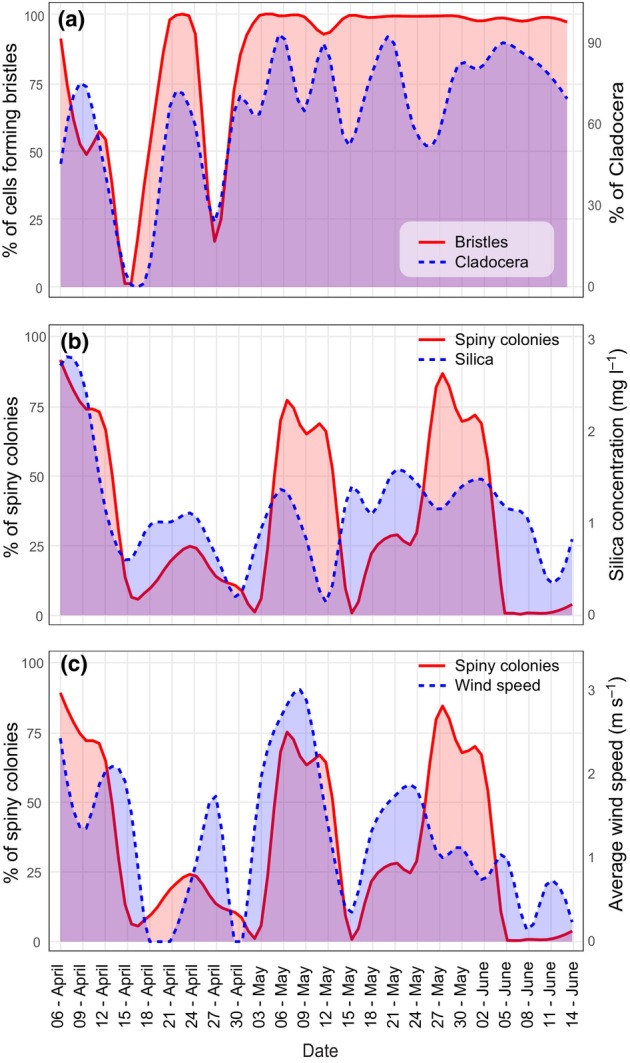
Most significant temporal correlations between morphological traits and environmental variables or zooplankton traits. These include (a) bristle formation with Cladocera relative abundance, (b) spiny colony relative abundance with silica concentration, and (c) spiny colony relative abundance with average wind speed.

The investigated Synurales phytoplankton community exhibited rapid changes not only in species composition but also in morphological traits (Fig. 3). These changes typically occurred very rapidly, within days, highlighting the need for studying phytoplankton on a finer temporal scale than the conventional monthly observations (Harris & Smith, 1977; Moreno‐Ostos *et al*., 2009). These rapid morphological shifts were correlated with both environmental data and zooplankton traits but differently for unicellular and colonial species. In unicellular species, we observed swift fluctuations in the presence of bristle‐forming species, which were clearly correlated with the abundance of cladoceran predators (Fig. 6a). This suggests that bristles serve as a defense mechanism against predation by this group of zooplankton. There are generally two hypotheses on the function of bristles. The first suggests that silica bristles evolved to aid in flotation, compensating for the inefficient vertical positioning of these small, weak‐swimming Chrysophytes (Naselli‐Flores & Barone, 2011; Siver, 2015). The second hypothesis posits that the bristles serve as a defense mechanism against predation since they make the cells difficult to ingest (Arvola *et al*., 1992; Kristiansen, 2005; Salonen *et al*., 2024). Our data strongly support the second hypothesis, showing that chemicals from grazing cladocerans induce the formation of spines in various phytoplankton groups (Hessen & van Donk, 1993; Lampert *et al*., 1994). Cladocera significantly influenced all evaluated bristle traits.

In the colonial species, the relationships with zooplankton were much weaker, indicating that their colonial lifestyle might be sufficient for defense against predation. Environmental factors, however, play a significant role in influencing their morphology (Figs 4a,b, 5b). The most important factors, correlated with the abrupt changes in colony shape, were average wind speed and silica levels (Fig. 6b,c). These likely reflect the presence of water mixing, resulting in the loss of stratification and an increase in nutrients from the bottom. Indeed, it is known that phytoplankton species are particularly sensitive to wind‐induced disturbances, which may modify the entire community (Margalef, 1978; Reynolds, 1993; Moreno‐Ostos *et al*., 2009). Such disturbances may potentially favor spiny species that depend more on silica for scale production. Indeed, laboratory studies confirmed that the species with spherical colonies and scales without spines might thrive even without scales, gaining a competitive advantage in silica‐limited conditions (Sandgren *et al*., 1996). This is in agreement with our morphological and genetic examination of scale‐less *Synura* colonial species (the so‐called *Syncrypta* morphotype), showing that species from the Petersenianae group, forming scales without the spines, occassionally occur in nature without scales (Pusztai & Škaloud, 2019). By contrast, species forming spiny, elongated colonies have so far never been found naked in nature.

Interestingly, while Pannard *et al*. (2008) hypothesized that increased mixing favored the largest phytoplankton size classes, we observed the opposite trend (Fig. 4c,d,h). It is possible that this effect applies to higher taxonomic units, and at the level of species, both cell and colony volume are rather dependent on biotic variables. In our case, low abundances of Cladocera and high abundances of Monogononta resulted in an increase in average cell and colony volume.

### Predator–prey interactions

During our investigation, Cladocera predominated among the predators in the samples and played a crucial role in shaping the community of unicellular species (Fig. 4e–g). Their abundance was negatively correlated with the abundance of Monogononta, leading to these two groups often showing apparently different correlations with the studied morphological traits (Fig. 4c,d,h). Interestingly, assignment to a higher taxonomic unit was the most significant variable in biotic data, while the influence of predator body size or feeding type was never significant. It is known that cladocerans are essential in filtration due to their body size (Downing & Peters, 1980; Haney, 1985) and capability to be very abundant in the water bodies (Sterner, 2009). Being the main filter feeders that can double the filtration rate during the night depending on the species (Starkweather, 1975; Haney, 1985), this can be up to hundreds of ml per day per individual (Smirnov, 2017). Without a doubt, the size of their body strongly affects the filtration rate (Egloff & Palmer, 1971) and the size of the food they can ingest (Burns, 1968; Geller & Müller, 1981). The size of detected Cladocera species ranged from 0.25 to 2.9 mm, with small species (Chydoridae) functioning mainly as secondary filter feeders (Gavrilko *et al*., 2020). Their method of feeding primarily involves scraping and collecting algae, bacteria, and detritus from surfaces (such as epipelon or macrophytes) and also filtering food particles from water (Fryer, 1968; Smirnov, 2017). Since unicellular species are smaller than colonial ones and often live in the epipelon due to their mixotrophic lifestyle, the presence of Chydoridae should affect them, but we did not observe this. Instead, we observed a clear relationship between cell and colony size and the presence of Monogononta, which are much smaller in size (their body size ranged from 0.14 to 0.83 mm).

It is possible that the size of Cladocera is already so large that defense against them by cell and colony size is ineffective, and the only strategy is the production of silica bristles, which can not only make ingestion difficult by increasing the prey's overall size but also may cause serious damage to predators. Indeed, the *Mallomonas* silica bristles are not only pointed but in most species are equipped with teeth (Fig. 1k,l), which may be created specifically to damage potential predators. Consequently, predators, although they could ingest them size‐wise, may learn to avoid these species and exclude them from their diet to prevent damage from bristles, both during ingestion and subsequent digestion. Interestingly, it is known that in cladocerans, as well as in other arthropods, the digestive tract is protected by a chitinous peritrophic membrane or peritrophic matrix, acting as a barrier against pathogens that enter the digestive tract orally (Gauld, 1957; Smirnov, 2017; Imaizumi *et al*., 2023). It is possible that this membrane may also effectively protect against damage by silica bristles, but this has not yet been investigated.

Conversely, increasing the volume of Synurales cells and colonies seems to be a suitable strategy against Monogononta predators, which are substantially smaller. These predators may then select other plankton particles as their food source, such as microalgae, heterotrophic protists, bacteria, detritus, etc. (Montemezzani *et al*., 2015; Gilbert, 2022). In addition, the formation of bristles as a damage‐causing strategy may be a nonfunctional strategy against Monogononta as they possess trophi, which are complex jaw‐like structures within their mastax capable of grinding, crushing, and cutting the bristles into small, harmless pieces (Hochberg *et al*., 2015).

### Environmental DNA surveys: the critical role of reference databases

eDNA surveys are a powerful tool for monitoring biodiversity, but their effectiveness heavily relies on the quality of reference databases. In microbial eukaryotes, the hyper‐variable V4 and V9 regions of the small subunit (SSU) rDNA are the most widely used markers for environmental sequencing (Dunthorn *et al*., 2012; Burki *et al*., 2021). Despite several advantages of focusing on SSU rDNA (Pawlowski *et al*., 2012), studies based on this marker face several challenges. First, many closely related species have SSU rDNA sequences that are very similar or even identical, making it impossible to distinguish them based on this sequence alone (Škaloud *et al*., 2015). Second, although sequenced in thousands of microbial species, reference SSU rDNA sequences are still missing for many others, making it often difficult to determine whether an undelimited OTU represents a genetically uncharacterized species, an undescribed lineage, or a chimeric sequence resulting from the PCR amplification process (Edgar *et al*., 2011). Accordingly, incomplete reference databases can often lead to artificial inflation of diversity estimates (Kunin *et al*., 2010; Fonseca *et al*., 2012).

In this study, we aimed to obtain as complete a reference database as possible to address all the above‐mentioned shortcomings. Since it is obviously not within human power to obtain sequences of all living phytoplankton species, we focused on one evolutionary lineage known for its significant role in freshwater ecosystems (Lengyel *et al*., 2023). In order to facilitate precise genetic identification of species, we obtained fast‐evolving ITS1 rDNA sequences of numerous cultured strains, encompassing all common species and 64% of taxa ever recorded in Europe. This uniquely complete reference database enabled us to assign virtually 100% of the obtained reads to individual species or taxonomically undescribed lineages. Accordingly, this has allowed us to precisely characterize the Synurales communities, distinguish between closely related species, minimize the risk of errors such as chimeric sequences, and assign morphological traits to each sequence obtained.

We would like to stress that high‐quality, comprehensive reference databases are essential for accurate data interpretation in many HTS studies. Today, as HTS technology is increasingly used in diversity studies, it is more important than ever to devote increased efforts to cultivating genetically uncharacterized protist species and lineages and to provide comprehensive morphological data along with sequences (del Campo *et al*., 2024).

### Conclusion

Our study demonstrates that using a comprehensive reference database allows us to translate diversity eDNA surveys into temporal changes in morphological diversity. These changes have been shown to occur very rapidly, within just days, meaning that key players with important ecological roles may not be recorded during a single observation. Our findings underscore the dynamic nature of freshwater ecosystems and the necessity for continuous monitoring to truly understand the intricate and ever‐changing abundances and interactions of species.

## Competing interests

None declared.

## Author contributions

PS and KT planned and designed the research. KT, RC, IJ and IC collected the data. PS ran the analyses with input from all co‐authors. PS wrote the first draft. PS, KT, RC, IJ and IC contributed to subsequent revisions of the paper. PS secured the funding.

## Disclaimer

The New Phytologist Foundation remains neutral with regard to jurisdictional claims in maps and in any institutional affiliations.

## Supporting information


**Fig. S1** Temporal variation of abiotic variables over 70 d of investigation.
**Fig. S2** Morphology of Synurales strains included in the ITS rDNA reference database.
**Fig. S3** The phylogeny of *Mallomonas* reference strains and swarms.
**Fig. S4** The phylogeny of *Synura* reference strains and swarms.
**Table S1** Summary of daily measurements of abiotic data.

**Table S2** Abundances of zooplankton species along with their functional traits.
**Table S3** Origin, sampling details, and ITS rDNA GenBank accession numbers of Synurales reference strains.

**Table S4** Abundances of Synurales species along with their functional traits.

**Table S5** Average functional traits across the entire community of algae and their predators.
Please note: Wiley is not responsible for the content or functionality of any Supporting Information supplied by the authors. Any queries (other than missing material) should be directed to the *New Phytologist* Central Office.

## Data Availability

The genetic data reported in this paper have been deposited in the National Center for Biotechnology Information (NCBI) Short Read Archive under the BioProject: PRJNA1217828. ITS rDNA sequences have been deposited in the NCBI, GenBank: PV036610–PV036701. Multiple alignments of organellar loci and ITS rDNA sequences are freely available on Mendeley Data: https://doi.org/10.17632/bpyvnrftbw.2.
