# Supplementary material for: High‐frequency sampling unveils biotic and abiotic drivers of rapid phytoplankton morphological changes
Source: New Phytol. 2025 Sep 4;248(5):2528–41. doi: 10.1111/nph.70534 (PMC12589706; doi:10.1111/nph.70534)
Supplement: Supplementary file 1 — Fig. S1 Temporal variation of abiotic variables over 70 d of investigation. Fig. S2 Morphology of Synurales strains included in the ITS rDNA reference database. Fig. S3 The phylogeny of Mallomonas reference strains and swarms. Fig. S4 The phylogeny of Synura reference strains and swarms. Table S1 Summary of daily measurements of abiotic data. [file NPH-248-2528-s004.docx]

## *New Phytologist* Supporting Information

Article title: **High-frequency sampling unveils biotic and abiotic drivers of rapid phytoplankton morphological changes**

Authors: Pavel Škaloud, Kateřina Tučková, Radka Čablová, Iva Jadrná and Ivana Černajová

Article acceptance date: 12 August 2025

The following Supporting Information is available for this article:

**Fig. S1** Temporal variation of abiotic variables over 70 days of investigation.

**Fig. S2** Morphology of Synurales strains included in the ITS rDNA reference database.

**Fig. S3** The phylogeny of *Mallomonas* reference strains and swarms.

**Fig. S4** The phylogeny of *Synura* reference strains and swarms.

**Table S1** Summary of daily measurements of abiotic data.

**Table S2** Abundances of zooplankton species, along with their functional traits.

**Table S3** Origin, sampling details and ITS rDNA GenBank accession numbers of Synurales reference strains.

**Table S4** Abundances of Synurales species, along with their functional traits.

**Table S5** Average functional traits across the entire community of algae and their predators.

**Fig. S1** Temporal variation of abiotic variables over 70 days of investigation, including precipitation (mm), sunshine duration (hours), calcium concentration (Ca, mg/L), average wind speed (m/s), total carbon (TC, mg/L), silicon concentration (Si, mg/L), total nitrogen (TN, mg/L), total phosphorus (TP, mg/L), pH, conductivity (µS/cm), water temperature (°C), and average daily air temperature (°C).

**
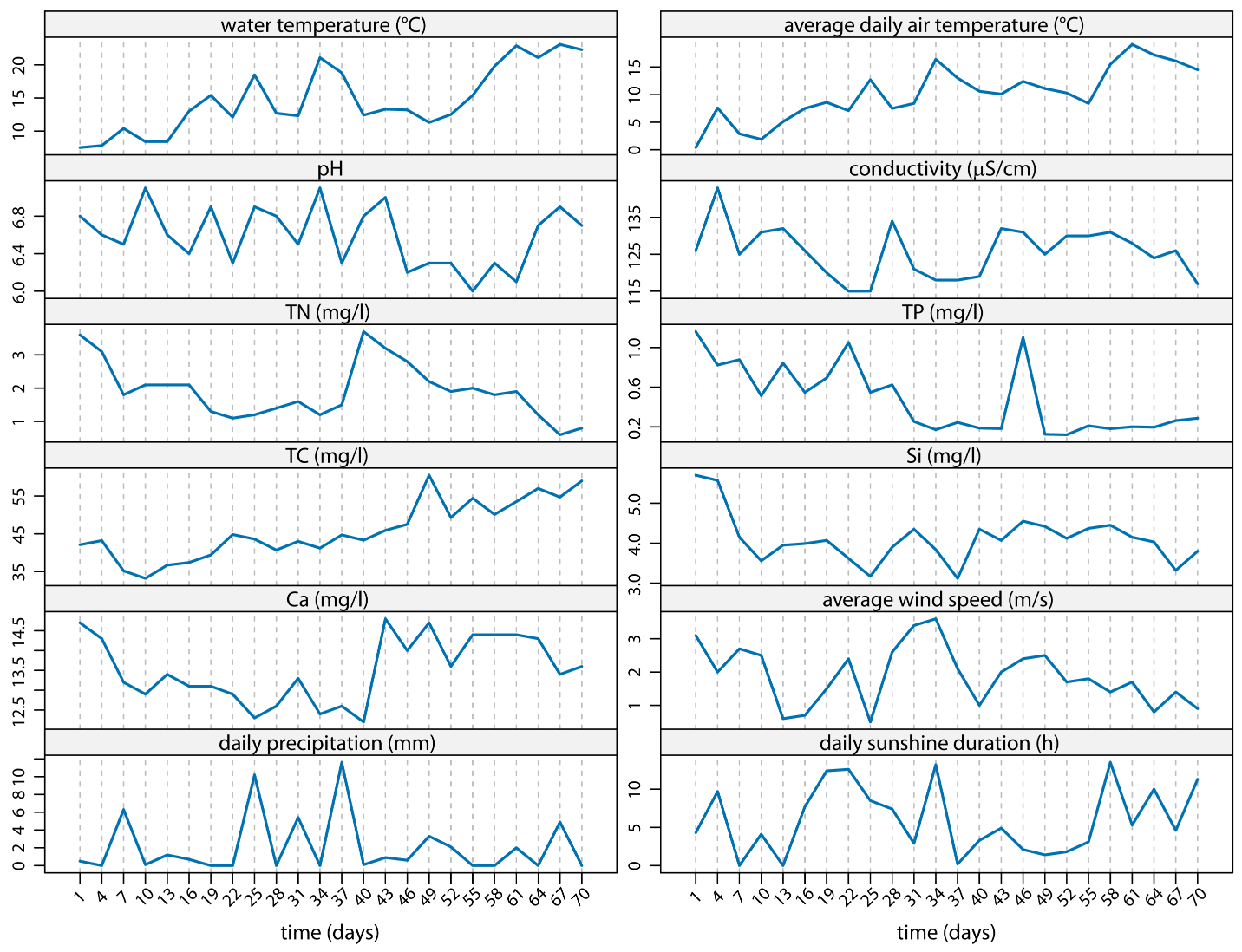
**

**Fig. S2** Morphology of Synurales strains included in the ITS rDNA reference database. Each strain is characterized by two silica scales examined under transmission electron microscopy (TEM), focusing on scales from both cultures (C) and field samples (F). If possible, different body scales are sown, including dome-bearing and domeless scales. Scale bars = 1 μm.

**
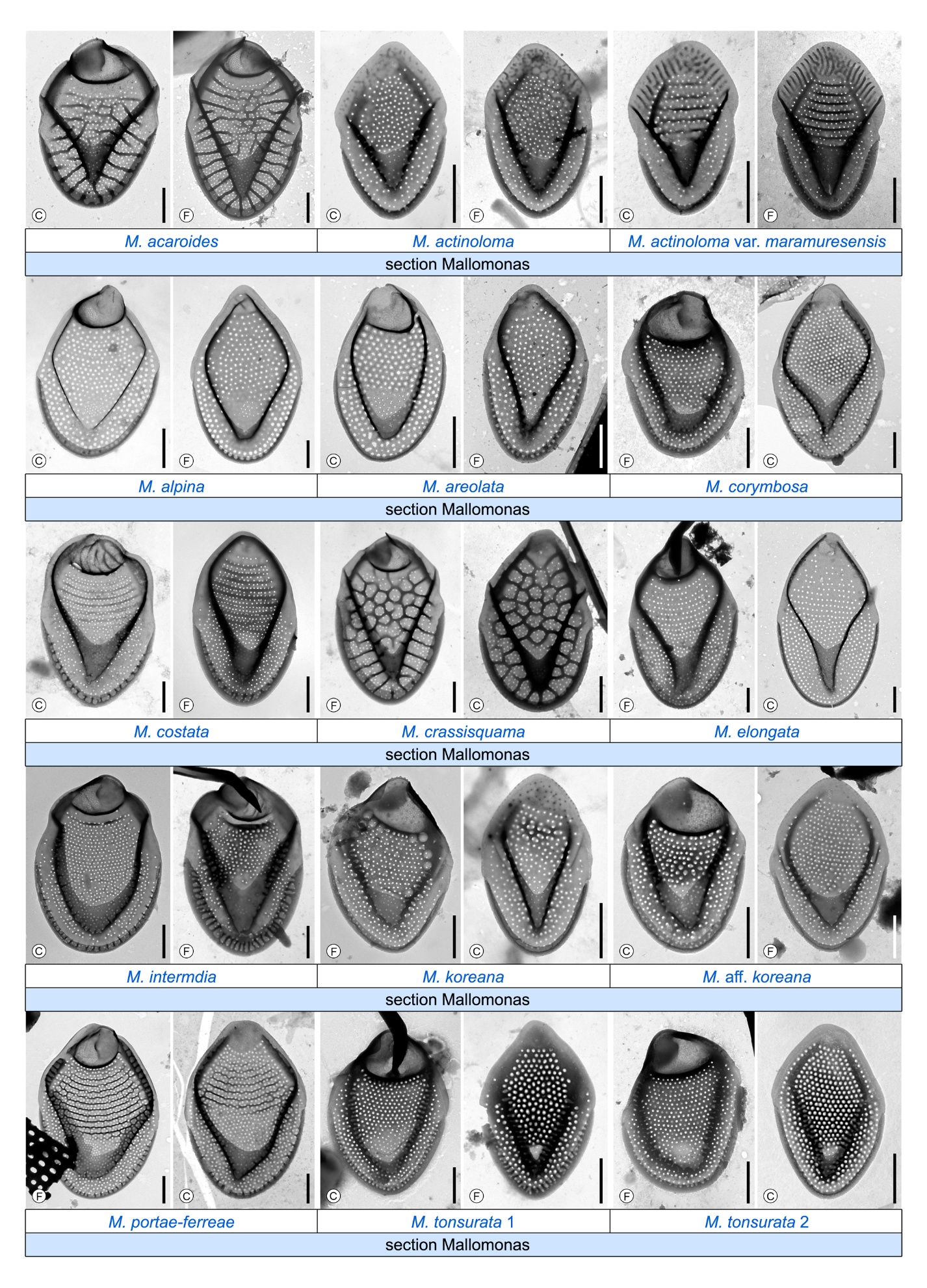
**

**
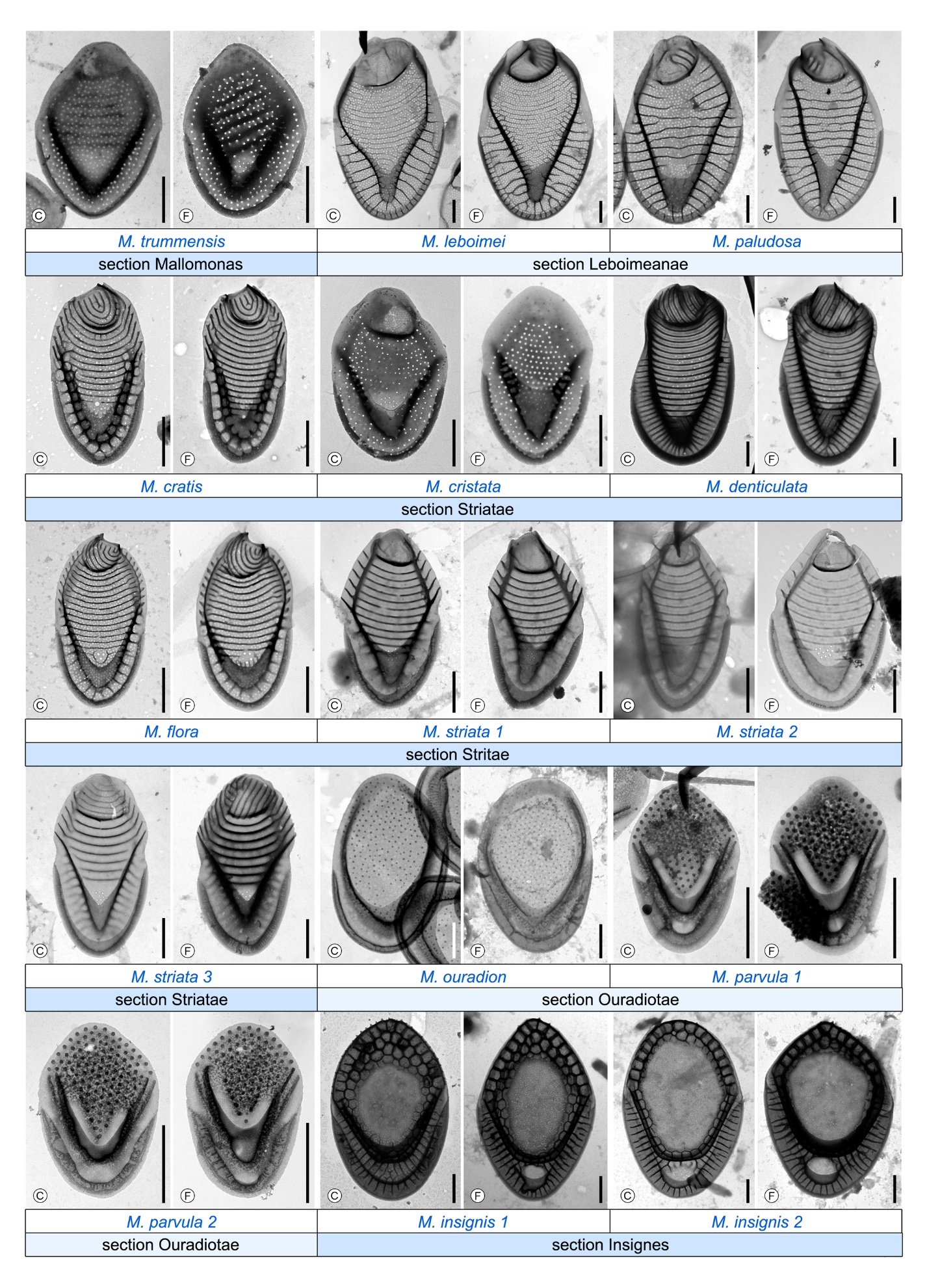
**

**
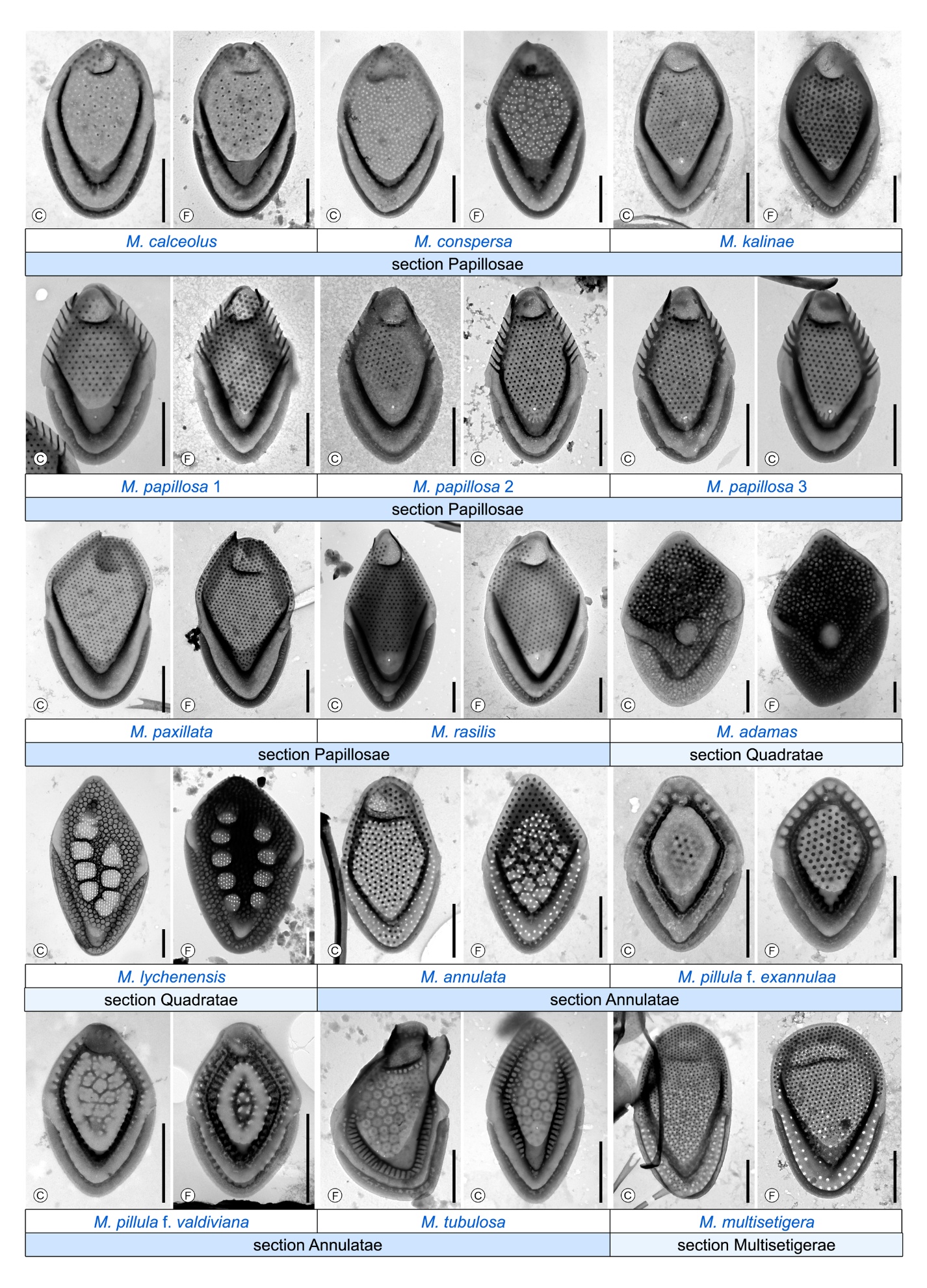
**

**
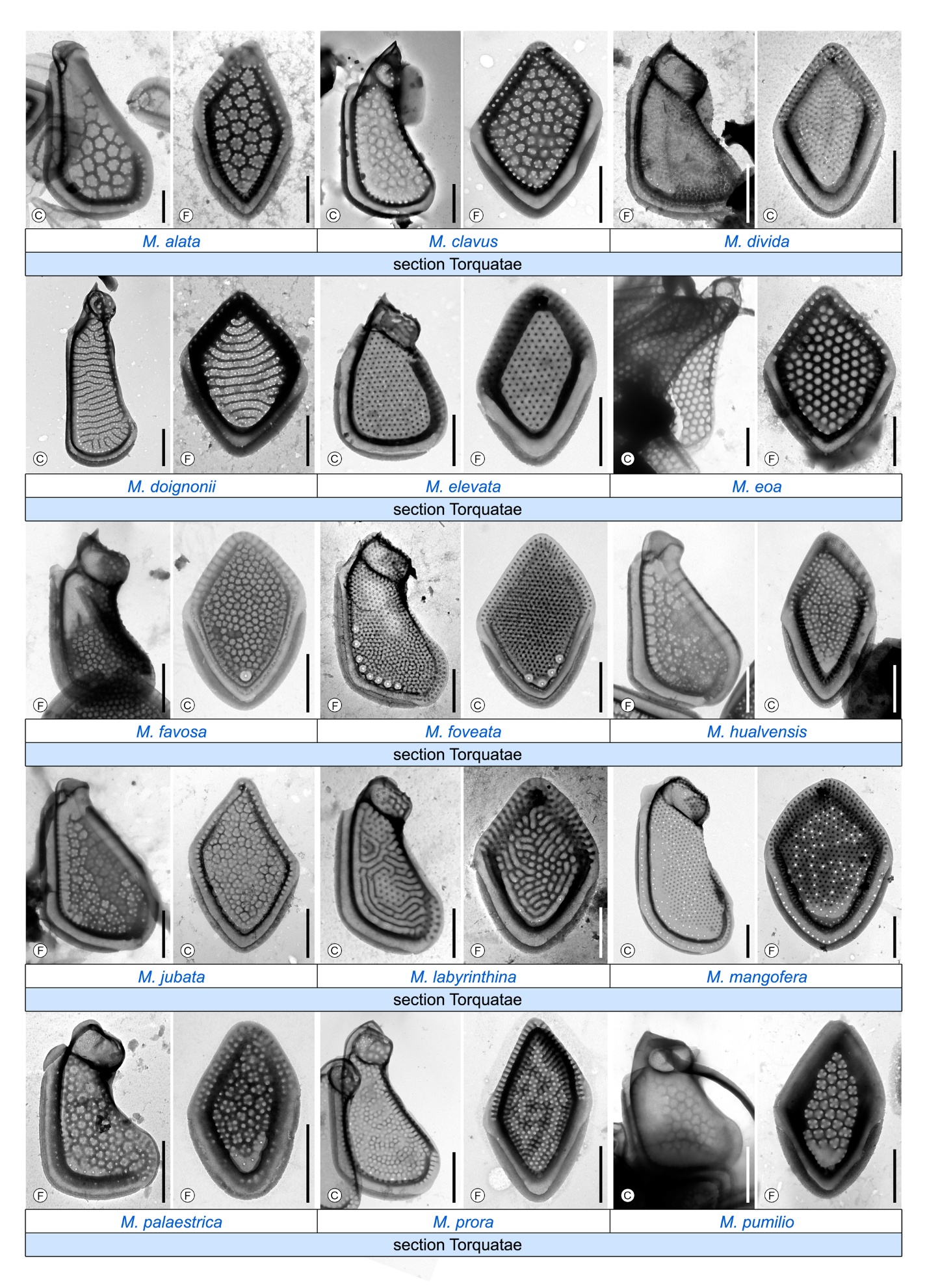
**

**
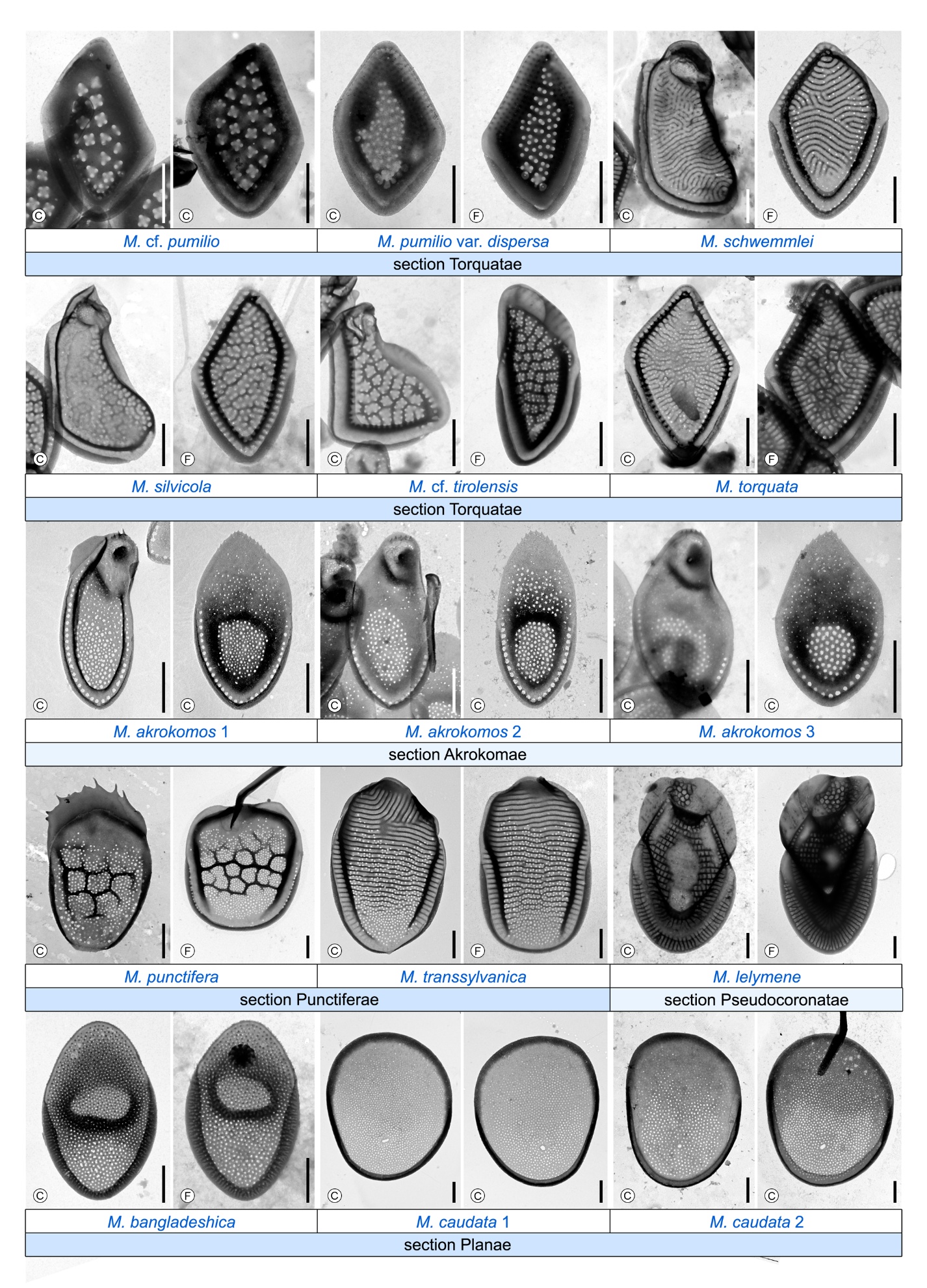
**

**
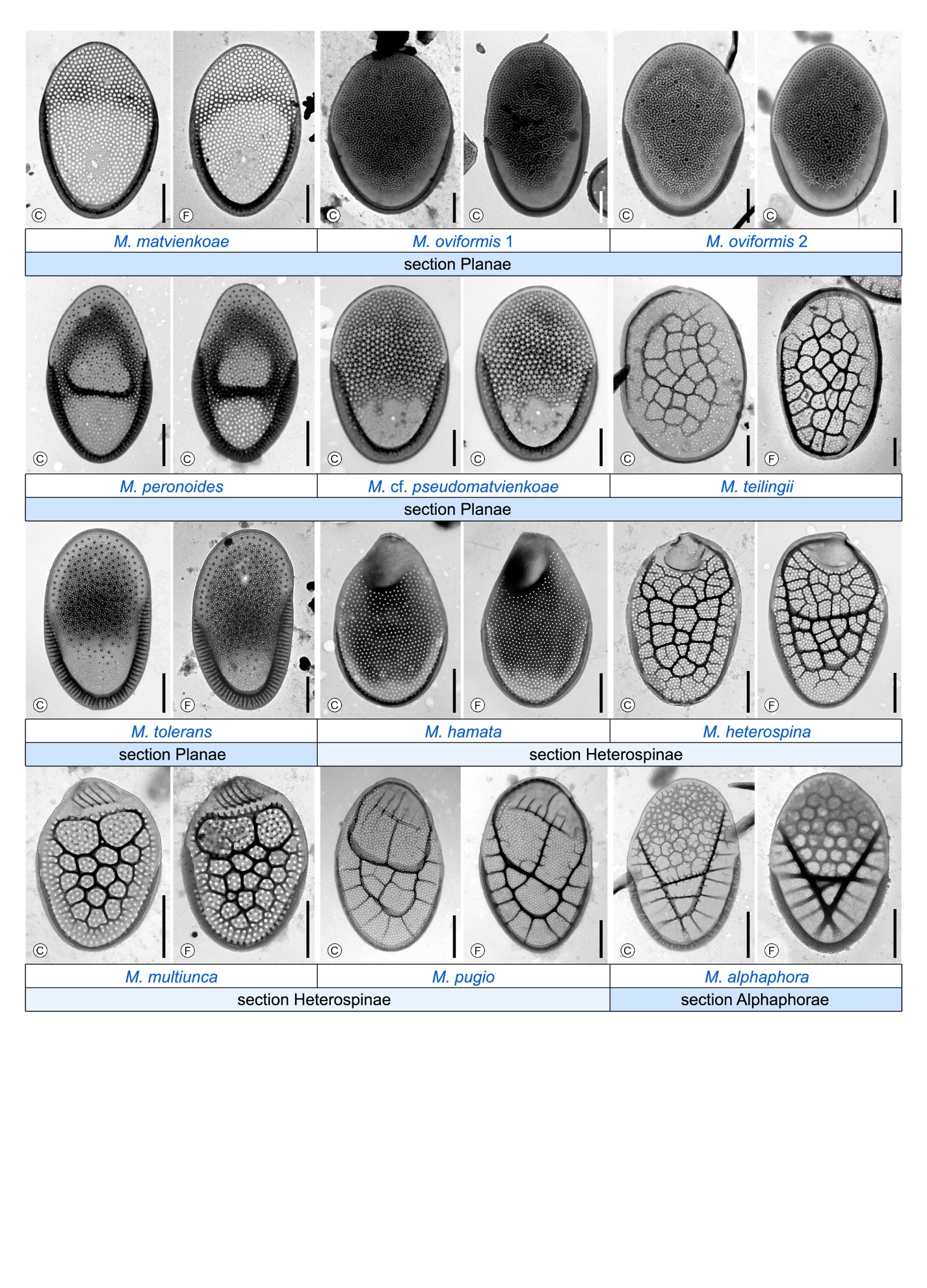
**

**
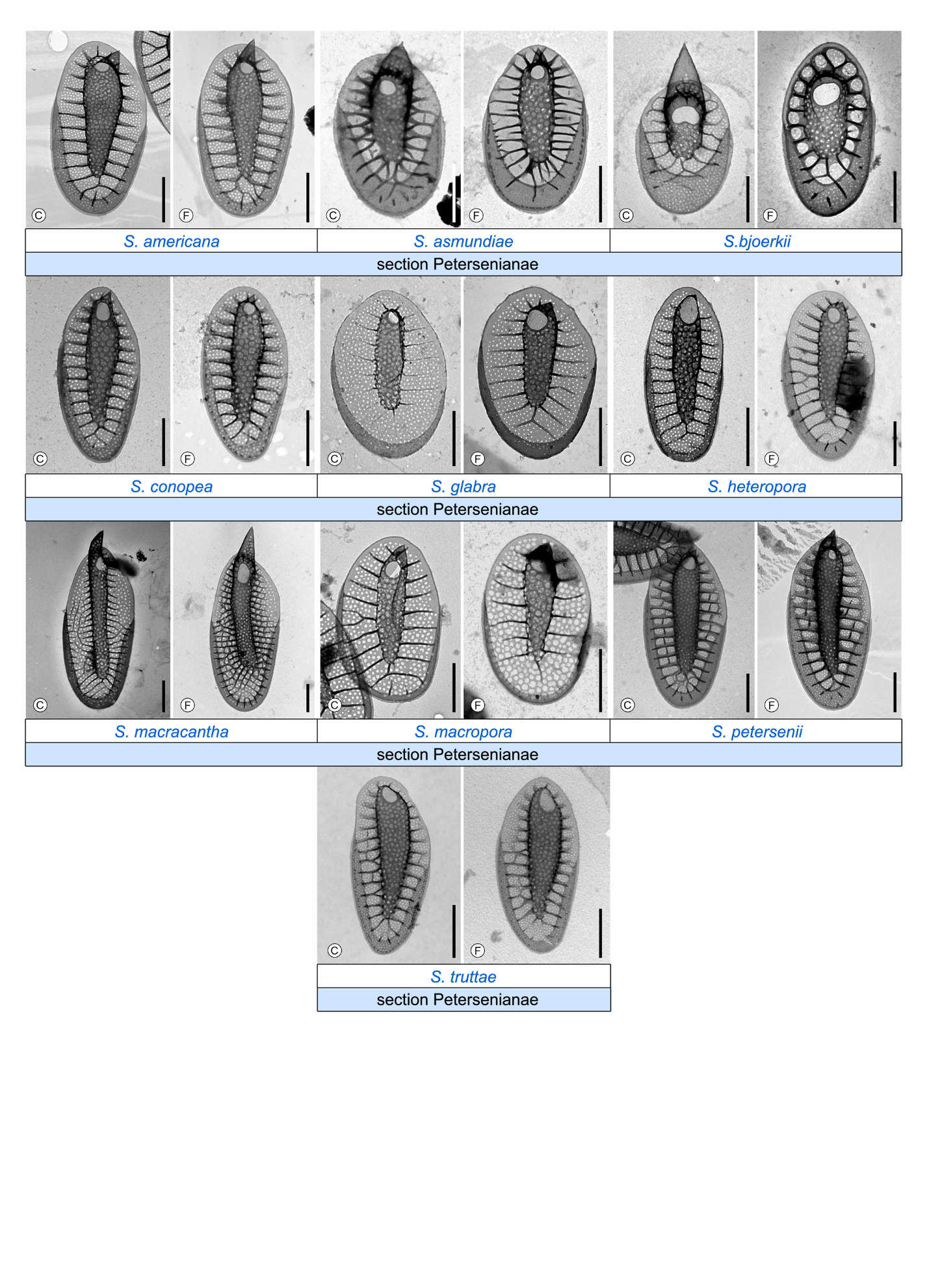
**

**
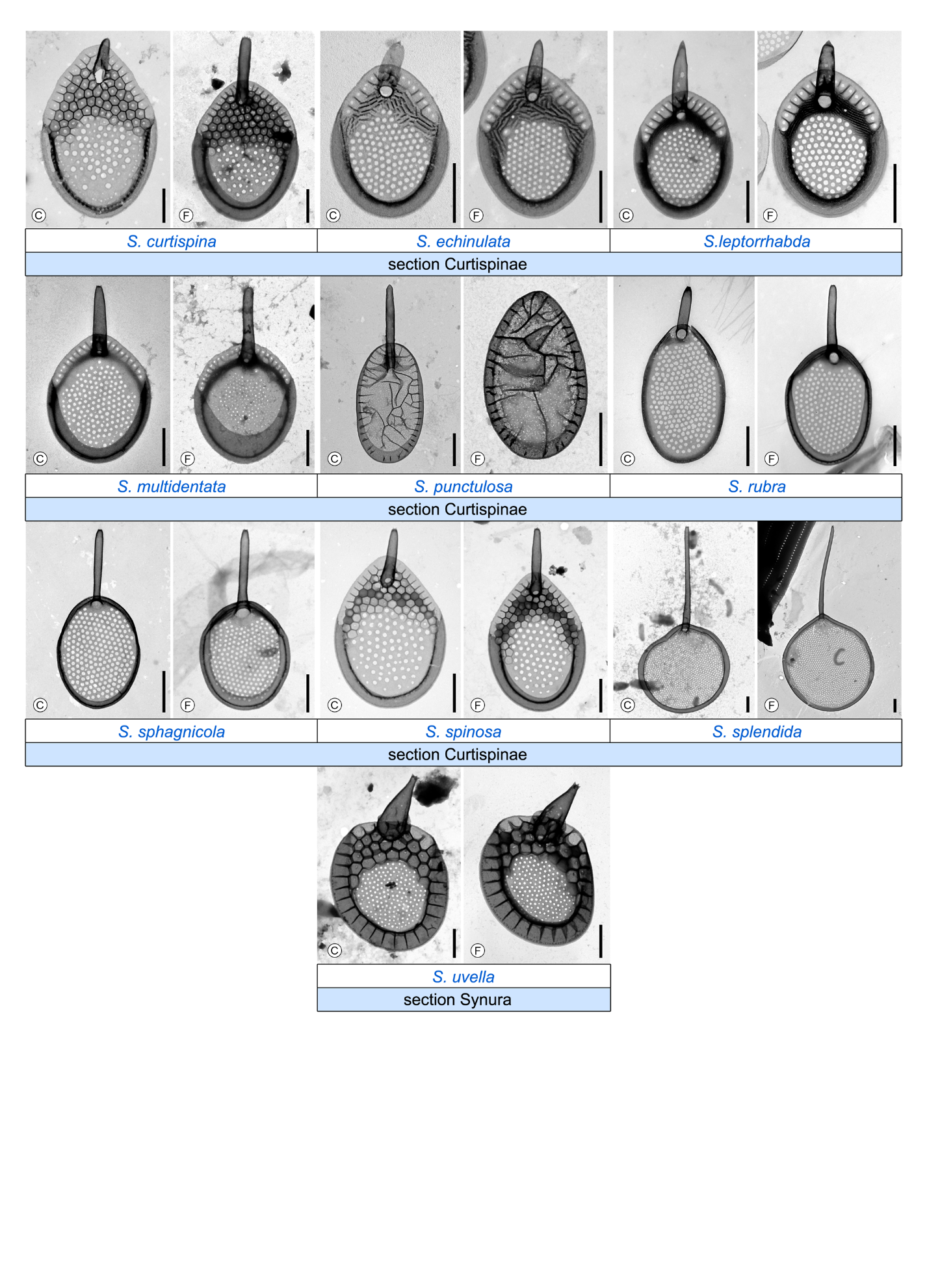
**

**Fig. S3** The phylogeny of *Mallomonas* reference strains and swarms based on the maximum likelihood (ML) analysis of ITS1 rDNA sequences. Values at the nodes indicate ML bootstrap values; only values higher than 70 are shown. Scale bar shows the estimated number of substitutions per site.

**
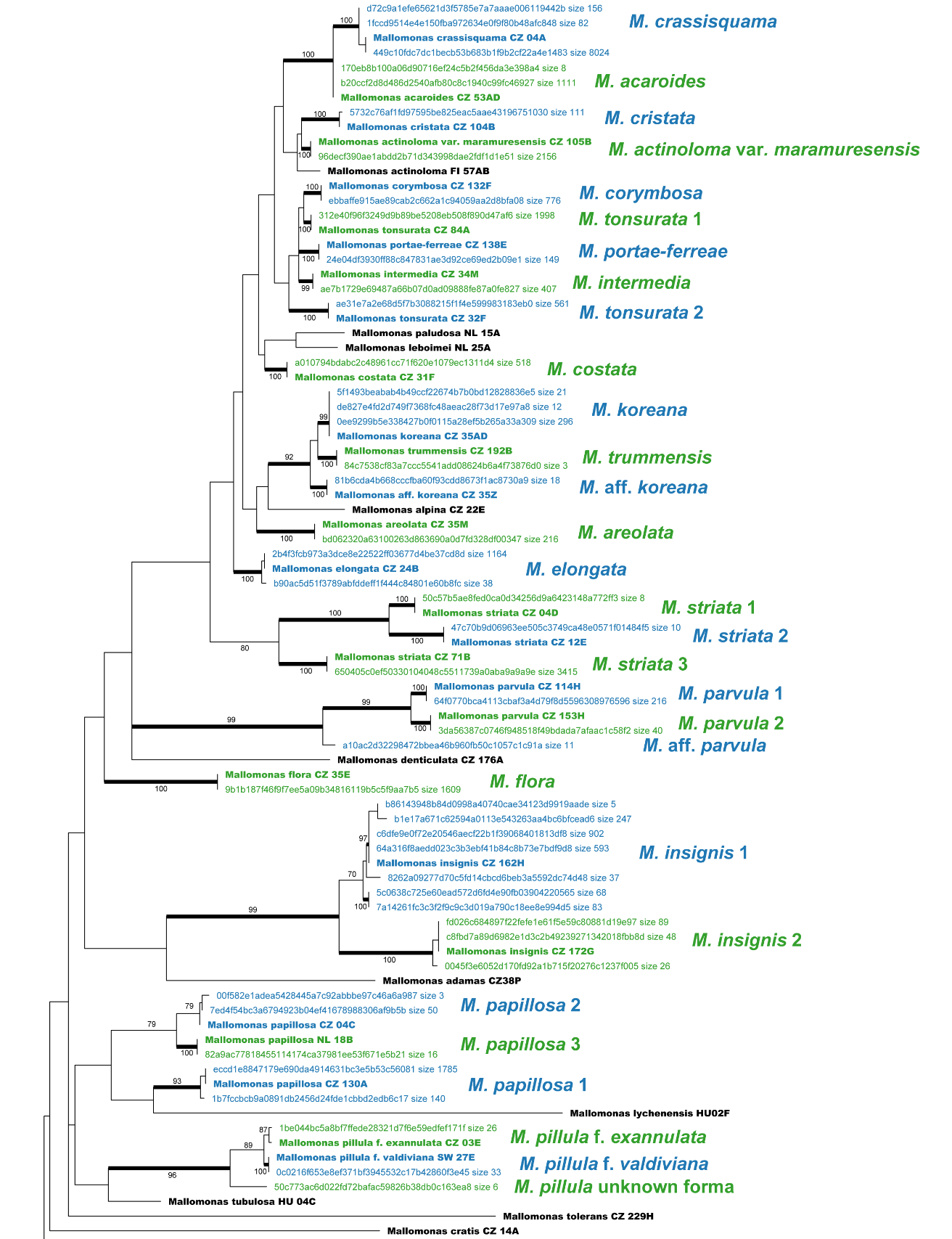
**

**
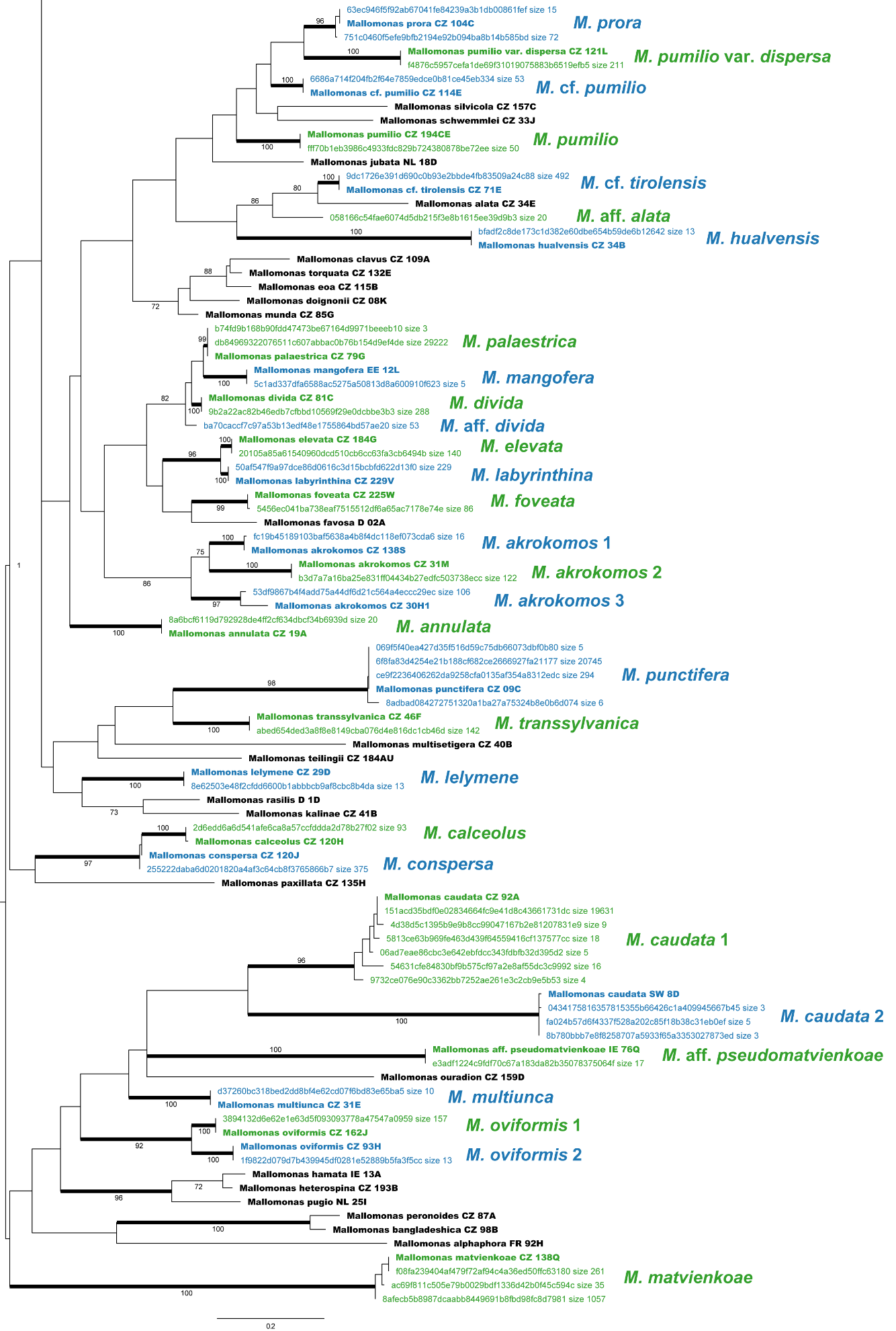
**

**Fig. S4** The phylogeny of *Synura* reference strains and swarms based on the maximum likelihood (ML) analysis of ITS1 rDNA sequences. Values at the nodes indicate ML bootstrap values; only values higher than 70 are shown. Scale bar shows the estimated number of substitutions per site.

**
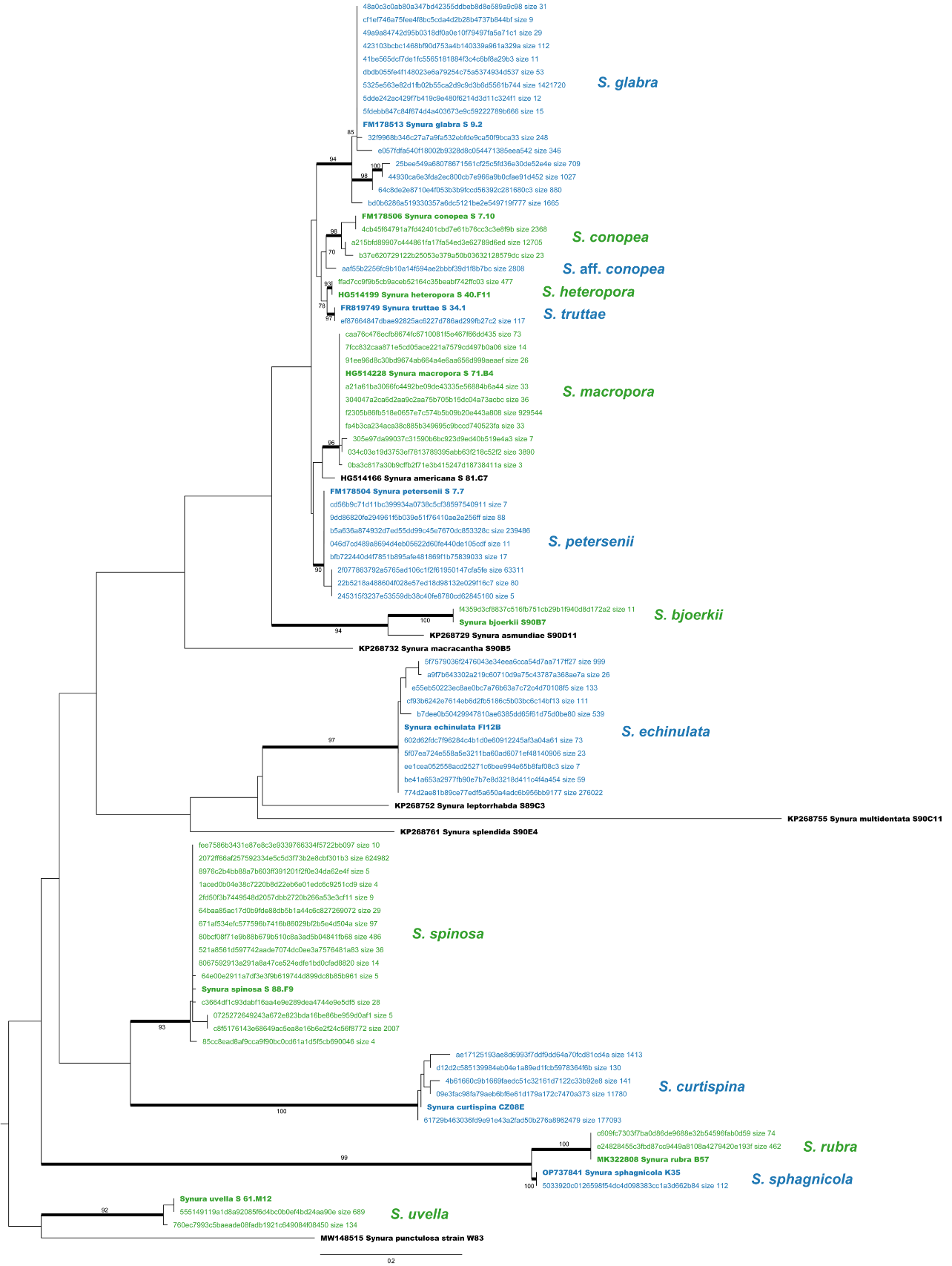
**

**Table S1** Summary of daily measurements of abiotic data.

| **date** | **time (days)** | **water temperature (°C)** | **average daily air temperature (°C)** | **maximum daily air temperature (°C)** | **minimum daily air temperature (°C)** | **pH** | **conductivity (µS/cm)** | **total nitrogen (TN, mg/l)** | **total phosphorus (TP, mg/l)** | **total carbon (TC, mg/l)** | **silica (Si, mg/l)** | **calcium (Ca, mg/l)** | **avg wind speed (m/s)** | **avg wind speed (m/s), day before** | **daily precipitation (mm)** | **daily precipitation (mm), day before** | **daily sunshine duration (h)** | **avg daily relative humidity (%)** |
| --- | --- | --- | --- | --- | --- | --- | --- | --- | --- | --- | --- | --- | --- | --- | --- | --- | --- | --- |
| **06.04.2021** | 1 | 7.5 | 0.4 | 5.1 | -3.9 | 6.8 | 126 | 3.6 | 1.16 | 42.1 | 5.7 | 14.7 | 3.1 | 2.7 | 0.5 | 1.7 | 4.3 | 71 |
| **09.04.2021** | 4 | 7.8 | 7.6 | 15.9 | -4.4 | 6.6 | 143 | 3.1 | 0.823 | 43.2 | 5.57 | 14.3 | 2 | 2.6 | 0 | 0.1 | 9.7 | 51 |
| **12.04.2021** | 7 | 10.4 | 2.9 | 12.1 | 0.4 | 6.5 | 125 | 1.8 | 0.877 | 35.2 | 4.15 | 13.2 | 2.7 | 1.4 | 6.3 | 0 | 0 | 87 |
| **15.04.2021** | 10 | 8.4 | 1.9 | 5.9 | -2.8 | 7.1 | 131 | 2.1 | 0.514 | 33.2 | 3.56 | 12.9 | 2.5 | 2.5 | 0.1 | 0 | 4.1 | 70 |
| **18.04.2021** | 13 | 8.4 | 5.1 | 8.8 | 2 | 6.6 | 132 | 2.1 | 0.843 | 36.7 | 3.95 | 13.4 | 0.6 | 1.7 | 1.2 | 0 | 0 | 84 |
| **21.04.2021** | 16 | 13 | 7.5 | 15.7 | 0.7 | 6.4 | 126 | 2.1 | 0.547 | 37.4 | 3.99 | 13.1 | 0.7 | 1.1 | 0.7 | 0.9 | 7.7 | 83 |
| **24.04.2021** | 19 | 15.4 | 8.6 | 16.2 | -2.1 | 6.9 | 120 | 1.3 | 0.693 | 39.4 | 4.07 | 13.1 | 1.5 | 1.4 | 0 | 0 | 12.4 | 57 |
| **27.04.2021** | 22 | 12.1 | 7.1 | 13.1 | -2.1 | 6.3 | 115 | 1.1 | 1.05 | 44.8 | 3.62 | 12.9 | 2.4 | 1.8 | 0 | 0 | 12.6 | 53 |
| **30.04.2021** | 25 | 18.5 | 12.7 | 19.6 | 9 | 6.9 | 115 | 1.2 | 0.547 | 43.6 | 3.17 | 12.3 | 0.5 | 2.9 | 10.2 | 1.4 | 8.5 | 67 |
| **03.05.2021** | 28 | 12.7 | 7.5 | 12.7 | 2.1 | 6.8 | 134 | 1.4 | 0.623 | 40.7 | 3.9 | 12.6 | 2.6 | 3 | 0 | 0.1 | 7.4 | 61 |
| **06.05.2021** | 31 | 12.3 | 8.4 | 13.5 | 1.3 | 6.5 | 121 | 1.6 | 0.254 | 43 | 4.35 | 13.3 | 3.4 | 2.9 | 5.4 | 1.8 | 2.9 | 69 |
| **09.05.2021** | 34 | 21.1 | 16.4 | 23.5 | 1.8 | 7.1 | 118 | 1.2 | 0.171 | 41.2 | 3.84 | 12.4 | 3.6 | 1.3 | 0 | 0 | 13.2 | 51 |
| **12.05.2021** | 37 | 18.8 | 13 | 21.3 | 11.9 | 6.3 | 118 | 1.5 | 0.245 | 44.7 | 3.12 | 12.6 | 2.1 | 3 | 11.6 | 3.8 | 0.2 | 94 |
| **15.05.2021** | 40 | 12.4 | 10.6 | 15.2 | 3.9 | 6.8 | 119 | 3.7 | 0.187 | 43.3 | 4.35 | 12.2 | 1 | 1 | 0.1 | 1.8 | 3.3 | 84 |
| **18.05.2021** | 43 | 13.3 | 10.1 | 15.9 | 5.3 | 7 | 132 | 3.2 | 0.182 | 45.9 | 4.07 | 14.8 | 2 | 2.1 | 0.9 | 1.4 | 4.9 | 73 |
| **21.05.2021** | 46 | 13.2 | 12.4 | 17.4 | 4.1 | 6.2 | 131 | 2.8 | 1.1 | 47.5 | 4.55 | 14 | 2.4 | 1.8 | 0.6 | 4.4 | 2.1 | 66 |
| **24.05.2021** | 49 | 11.3 | 11.1 | 17.1 | 2.8 | 6.3 | 125 | 2.2 | 0.125 | 60.5 | 4.42 | 14.7 | 2.5 | 1.1 | 3.3 | 3.1 | 1.4 | 78 |
| **27.05.2021** | 52 | 12.5 | 10.3 | 16.2 | 8 | 6.3 | 130 | 1.9 | 0.12 | 49.3 | 4.12 | 13.6 | 1.7 | 3.1 | 2.1 | 0 | 1.8 | 72 |
| **30.05.2021** | 55 | 15.4 | 8.4 | 12.7 | 5 | 6 | 130 | 2 | 0.211 | 54.4 | 4.37 | 14.4 | 1.8 | 1.7 | 0 | 0.1 | 3.1 | 78 |
| **02.06.2021** | 58 | 19.8 | 15.5 | 22.8 | 4.6 | 6.3 | 131 | 1.8 | 0.181 | 50.1 | 4.45 | 14.4 | 1.4 | 0.8 | 0 | 0 | 13.5 | 56 |
| **05.06.2021** | 61 | 22.9 | 19.1 | 28.2 | 13.7 | 6.1 | 128 | 1.9 | 0.201 | 53.5 | 4.15 | 14.4 | 1.7 | 1.1 | 2 | 0 | 5.3 | 71 |
| **08.06.2021** | 64 | 21.1 | 17.2 | 25.7 | 10.2 | 6.7 | 124 | 1.2 | 0.196 | 57 | 4.03 | 14.3 | 0.8 | 0.5 | 0 | 0 | 10 | 69 |
| **11.06.2021** | 67 | 23.1 | 16.1 | 24.7 | 9.9 | 6.9 | 126 | 0.6 | 0.263 | 54.7 | 3.32 | 13.4 | 1.4 | 0.9 | 4.9 | 0.8 | 4.6 | 86 |
| **14.06.2021** | 70 | 22.3 | 14.5 | 22.4 | 5.4 | 6.7 | 117 | 0.8 | 0.287 | 59 | 3.8 | 13.6 | 0.9 | 2.7 | 0 | 0 | 11.3 | 66 |

**Table S2** The absolute numbers and relative abundances of identified zooplankton species, along with their functional traits. Major taxonomic groups (Arthropoda-Cladocera, Arthropoda-Copepoda and Rotifera) are colour-coded for clarity.

*Provided as a separate file.*

**Table S3** Origin, sampling details and ITS rDNA GenBank accession numbers of Synurales reference strains. Newly generated sequences are given in bold.

| **taxon** | **strain** | **collection site** | **sampling date** | **coordinates** | **t (°C)** | **pH** | **c (µS/cm)** | **ITS rDNA GenBank accessions** |
| --- | --- | --- | --- | --- | --- | --- | --- | --- |
| *M. acaroides* | CZ 53A-D | Czechia, Malé Dářko pond | 11.05.2020 | 49.6644739, 15.8800561 | 17.7 | 8.14 | 150 | **PV036610** |
| *M. actinoloma* | FI 57AB | Finland, Keihäsjärvi lake | 14.05.2023 | 62.1092311, 23.7180581 | 17.5 | 5.6 | 41 | **PV036611** |
| *M. actinoloma* var*. maramuresensis* | CZ 105B | Czechia, V Rašeliništích pond | 30.10.2020 | 48.7116014, 14.2037072 | 7.5 | 6 | 76 | **PV036612** |
| *M. adamas* | CZ 38P | Czechia, Mariánský pond | 04.05.2020 | 50.54551, 14.67669 | 10.8 | 6.4 | 121 | **PV036613** |
| *M. akrokomos* | CZ 138S | Czechia, Morava oxbow river | 08.11.2020 | 48.8027053, 17.0940214 | 9.3 | 7.3 | 372 | **PV036614** |
| *M. akrokomos* | CZ 31M | Czechia, alluvial pool Drnků | 27.04.2020 | 48.85259, 14.9087 | 13.6 | 6.9 | 238 | **PV036615** |
| *M. akrokomos* | CZ 30H1 | Czechia, forest pond near Zásada | 17.04.2020 | 50.6925339, 15.2629731 | - | 6.5 | 98 | **PV036616** |
| *M. alata* | CZ 34E | Czechia, Lužnice oxbow lake, Suchdol | 27.04.2020 | 48.9088967, 14.8907903 | 13.8 | 7.1 | 207 | **PV036617** |
| *M. alphaphora* | FR 92H | France, swamp near Lac de Hourtin | 29.03.2023 | 45.0600275, -1.1198053 | 13.7 | 7.6 | 310 | **PV036618** |
| *M. alpina* | CZ 22E | Czechia, Tubož pond | 12.04.2020 | 50.4905461, 14.5997953 | 15 | 8.5 | 470 | **PV036619** |
| *M. annulata* | CZ 19A | Czechia, Křtiny pond | 06.04.2020 | 50.0256500, 14.5350828 | 12 | 7.6 | 1052 | **PV036620** |
| *M. areolate* | CZ 35M | Czechia, Zlatá stoka canal | 27.04.2020 | 48.9518128, 14.8681525 | 12.2 | 7.7 | 288 | **PV036621** |
| *M. bangladeshica* | CZ 98B | Czechia, Odra oxbow lake, Zábřeh | 10.10.2020 | 49.8082639, 18.2179922 | 13.6 | 7.4 | 368 | **PV036622** |
| *M. calceolus* | CZ 120H | Czechia, Farský pond | 06.11.2020 | 49.5586869, 15.9071714 | 6.7 | 6.6 | 117 | **PV036623** |
| *M. caudata* | CZ 92A | Czechia, Kurfürstovo rameno oxbow lake | 08.10.2020 | 49.6612536, 17.2125003 | 12.5 | 7.3 | 698 | **PV036624** |
| *M. caudata* | SW 08D | Sweden, Klövabäcken stream | 30.05.2021 | 56.0658369, 13.1224364 | 14.6 | 6.1 | 99 | **PV036625** |
| *M. clavus* | CZ 109A | Czechia, pond on Smrčinský brook | 30.10.2020 | 48.7689736, 13.9644942 | 7.8 | 6.4 | 42 | **PV036626** |
| *M. conspersa* | CZ 120J | Czechia, Farský pond | 06.11.2020 | 49.5586869, 15.9071714 | 6.7 | 6.6 | 117 | **PV036627** |
| *M. corymbose* | CZ 132F | Czechia, canal, Soutok | 08.11.2020 | 48.6628856, 16.9375286 | 8.7 | 7.5 | 908 | **PV036628** |
| *M. costata* | CZ 31F | Czechia, alluvial pool Drnků | 27.04.2020 | 48.85259, 14.9087 | 13.6 | 6.9 | 238 | **PV036629** |
| *M. crassisquama* | CZ 04A | Czechia, Malše river | 24.02.2020 | 48.975711, 14.470721 | 4 | 8.4 | 163 | **PV036630** |
| *M. cratis* | CZ 14A | Czechia, Vltava river | 04.04.2020 | 50.1336267, 14.3935644 | 8.1 | 9.7 | 337 | **PV036631** |
| *M. cristata* | CZ 104B | Czechia, pool Na Plachtě | 26.10.2020 | 50.1886000, 15.8607278 | 12.1 | 7 | 51 | **PV036632** |
| *M. denticulata* | CZ 176A | Czechia, pond on Suchá Bělá | 27.04.2021 | 50.8810533, 14.2610569 | 10 | 6.1 | 92 | **PV036633** |
| *M. divida* | CZ 81C | Czechia, Zlatá stoka canal | 16.09.2020 | 48.9814172, 14.7943708 | 17.5 | 7.2 | 194 | **PV036634** |
| *M. doignonii* | CZ 08K | Czechia, alluvial pool Drnků | 12.03.2020 | 48.85259, 14.9087 | 10.5 | 8.2 | 228 | **PV036635** |
| *M. elevate* | CZ 184G | Czechia, Spálená Borkovna peat bog | 06.01.2022 | 48.9809317, 14.8000531 | 3.4 | 6.8 | 149 | **PV036636** |
| *M. elongata* | CZ 24B | Czechia, Kačírek pond | 12.04.2020 | 50.4361561, 14.5805639 | 15 | 8.4 | 405 | **PV036637** |
| *M. eoa* | CZ 115B | Czechia, Kladský pond | 31.10.2020 | 50.0277756, 12.6750450 | 7.6 | 5.9 | 49 | **PV036638** |
| *M. favosa* | D 02A | Denmark, Bornholm, Krystalso | 09.09.2020 | 55.2845044, 14.7584786 | - | - | - | **PV036639** |
| *M. flora* | CZ 35E | Czechia, Zlatá stoka canal | 27.04.2020 | 48.9518128, 14.8681525 | 12.2 | 7.7 | 288 | **PV036640** |
| *M. foveata* | CZ 225W | Czechia, Spálená Borkovna peat bog | 26.10.2023 | 48.9809317, 14.8000531 | 11.5 | 6.3 | 130 | **PV036641** |
| *M. hamata* | IE 13A | Ireland, Muckross Lake | 13.10.2021 | 52.0056528, -9.5298981 | 14.4 | 6.9 | 51 | **PV036642** |
| *M. heterospina* | CZ 193B | Czechia, alluvial pool 107 | 10.03.2022 | 48.8391514, 14.9149114 | 4.3 | 7.7 | 278 | **PV036643** |
| *M. hualvensis* | CZ 34B | Czechia, Lužnice oxbow lake, Suchdol | 27.04.2020 | 48.9088967, 14.8907903 | 13.8 | 7.1 | 207 | **PV036644** |
| *M. insignis* | CZ 172G | Czechia, Eiland pond | 27.04.2021 | 50.8020389, 14.0465794 | 13.2 | 7.2 | 89 | **PV036645** |
| *M. insignis* | CZ 162H | Czechia, Spálená Borkovna peat bog | 07.04.2021 | 48.9809317, 14.8000531 | 7.5 | 6.8 | 320 | **PV036646** |
| *M. intermedia* | CZ 34M | Czechia, Lužnice oxbow lake, Suchdol | 27.04.2020 | 48.9088967, 14.8907903 | 13.8 | 7.1 | 207 | **PV036647** |
| *M. jubata* | NL 18D | Netherlands, unnamed pond in Plasmolen | 13.11.2021 | 51.7380808, 5.9300058 | 10.3 | 7.6 | 352 | **PV036648** |
| *M. kalinae* | CZ 41B | Czechia, Swamp peat bog | 04.05.2020 | 50.5948858, 14.6431294 | 11 | 7.3 | 182 | **PV036649** |
| *M. koreana* | CZ 35AD | Czechia, Zlatá stoka canal | 27.04.2020 | 48.9518128, 14.8681525 | 12.2 | 7.7 | 288 | **PV036650** |
| *M.* aff*. koreana* | CZ 35Z | Czechia, Zlatá stoka canal | 27.04.2020 | 48.9518128, 14.8681525 | 12.2 | 7.7 | 288 | **PV036651** |
| *M. labyrinthina* | CZ 229V | Czechia, Spálená Borkovna peat bog | 29.10.2023 | 48.9807556, 14.8003000 | 12 | 5.6 | 263 | **PV036652** |
| *M. leboimei* | NL 25A | Netherlands, Peetersven lake | 14.11.2021 | 51.3678672, 5.4666636 | 9.6 | 5.3 | 28 | **PV036653** |
| *M. lelymene* | CZ 29D | Czechia, Jirkov quarry | 17.04.2020 | 50.6650733, 15.2761828 | - | 6 | 46 | **PV036654** |
| *M. lychenensis* | HU 02F | Hungary, Risztics parlag | 15.04.2022 | 47.7058658, 19.0426764 | 10.8 | 6.8 | 173 | **PV036655** |
| *M. mangofera* | EE 12L | Estonia, Halliste jögi oxbow lake | 11.05.2023 | 58.3631903, 25.0601917 | 14.6 | 7.6 | 431 | **PV036656** |
| *M. matvienkoae* | CZ 138Q | Czechia, Morava oxbow river | 08.11.2020 | 48.8027053, 17.0940214 | 9.3 | 7.3 | 372 | **PV036657** |
| *M. multisetigera* | CZ 40B | Czechia, Swamp peat bog | 04.05.2020 | 50.5760483, 14.6696636 | 12.8 | 6.2 | 79 | **PV036658** |
| *M. multiunca* | CZ 31E | Czechia, alluvial pool Drnků | 27.04.2020 | 48.85259, 14.9087 | 13.6 | 6.9 | 238 | **PV036659** |
| *M. ouradion* | CZ 159D | Czechia, forest pond Gabom | 11.12.2020 | 49.0001461, 14.8128283 | 3.8 | 5.2 | 98 | **PV036660** |
| *M. oviformis* | CZ 93H | Czechia, wetland, Horka nad Moravou | 08.10.2020 | 49.6410397, 17.2019122 | 13.2 | 7.5 | 515 | **PV036661** |
| *M. oviformis* | CZ 162J | Czechia, Spálená Borkovna peat bog | 07.04.2021 | 48.9809317, 14.8000531 | 7.5 | 6.8 | 320 | **PV036662** |
| *M. palaestrica* | CZ 79G | Czechia, Podřezanská canal | 16.09.2020 | 48.9806342, 14.7969525 | 20.3 | 6.7 | 137 | **PV036663** |
| *M. paludosa* | NL 15A | Netherlands, Botersnijder West lake | 13.11.2021 | 51.7828019, 5.7992242 | 9.5 | 6 | 113 | **PV036664** |
| *M. papillosa* | CZ 130A | Czechia, pond near Dyje rver | 07.11.2020 | 48.8229353, 16.7825411 | 8.3 | 7.1 | 567 | **PV036665** |
| *M. papillosa* | CZ 04C | Czechia, Malše river | 24.02.2020 | 48.975711, 14.470721 | 4 | 8.4 | 163 | **PV036666** |
| *M. papillosa* | NL 18B | Netherlands, unnamed pond in Plasmolen | 13.11.2021 | 51.7380808, 5.9300058 | 10.3 | 7.6 | 352 | **PV036667** |
| *M. parvula* | CZ 114H | Czechia, forest pond | 31.10.2020 | 49.9800311, 12.7870547 | 9 | 6.6 | 320 | **PV036668** |
| *M. parvula* | CZ 153H | Czechia, Spálená Borkovna peat bog | 11.12.2020 | 48.9804136, 14.8003006 | 3.2 | 6.2 | 159 | **PV036669** |
| *M. paxillata* | CZ 135H | Czechia, Vlčí struha canal, Soutok | 08.11.2020 | 48.6618158, 16.9557658 | 8.6 | 7.3 | 528 | **PV036670** |
| *M. peronoides* | CZ 87A | Czechia, Podřezanská canal | 09.10.2020 | 48.9806342, 14.7969525 | 13.1 | 6.7 | 153 | **PV036671** |
| *M. pillula* f. *exannulata* | CZ 03E | Czechia, Podhorní pond | 13.02.2020 | 50.1423467, 13.8892633 | 2.1 | 7.8 | 512 | **PV036672** |
| *M. pillula* f. *valdiviana* | SW 27E | Sweden, unnamed small river | 01.06.2021 | 57.2664808, 13.1179544 | 18.7 | 6 | 61 | **PV036673** |
| *M. portae-ferreae* | CZ 138E | Czechia, Morava oxbow river | 08.11.2020 | 48.8027053, 17.0940214 | 9.3 | 7.3 | 372 | **PV036674** |
| *M. prora* | CZ 104C | Czechia, pool Na Plachtě | 26.10.2020 | 50.1886000, 15.8607278 | 12.1 | 7 | 51 | **PV036675** |
| *M.* cf. *pseudomatvienkoae* | IE 76Q | Ireland, Lake near Peacocke's | 10.11.2023 | 53.4565864, -9.5420106 | 8.1 | 4.88 | 53.1 | **PV036676** |
| *M. pugio* | NL 25I | Netherlands, Peetersven lake | 14.11.2021 | 51.3678672, 5.4666636 | 9.6 | 5.3 | 28 | **PV036677** |
| *M. pumilio* | CZ 194C | Czechia, Dolní Trocnov pond | 11.03.2022 | 48.8917042, 14.6090925 | 4.8 | 8 | 136 | **PV036678** |
| *M.* cf. *pumilio* | CZ 114E | Czechia, forest pond | 31.10.2020 | 49.9800311, 12.7870547 | 9 | 6.6 | 320 | **PV036679** |
| *M. pumilio* var. *dispersa* | CZ 121L | Czechia, Fejtovský pond | 06.11.2020 | 49.6182897, 15.9021958 | 9.4 | 4.4 | 97 | **PV036680** |
| *M. punctifera* | SW 52C | Sweden, Lygnern lake | 04.06.2021 | 57.5348617, 12.4288819 | 15.5 | 6.4 | 85 | **PV036681** |
| *M. rasilis* | D 01D | Denmark, Bornholm, Svinemose | 08.09.2020 | 55.1086511, 14.9487119 | 15.4 | 6.72 | 516 | **PV036682** |
| *M. schwemmlei* | CZ 33J | Czechia, Lužnice oxbow lake, Dvory | 27.04.2020 | 48.8564053, 14.8993053 | 13.2 | 7.8 | 130 | **PV036683** |
| *M. silvicola* | CZ 157C | Czechia, forest pond Congo | 11.12.2020 | 49.0003089, 14.8128069 | 3.9 | 5.8 | 31 | **PV036684** |
| *M. striata* | CZ 04D | Czechia, Malše river | 24.02.2020 | 48.975711, 14.470721 | 4 | 8.4 | 163 | **PV036685** |
| *M. striata* | CZ 12E | Czechia, alluvial pool 207 | 13.03.2020 | 48.86438, 14.89414 | 10.2 | 7.9 | 179 | **PV036686** |
| *M. striata* | CZ 71B | Czechia, Svratka pond | 18.05.2020 | 49.7069431, 16.0270547 | 12.2 | 6.8 | 114 | **PV036687** |
| *M. teilingii* | CZ 184AU | Czechia, Spálená Borkovna peat bog | 06.01.2022 | 48.9809317, 14.8000531 | 3.4 | 6.8 | 149 | **PV036688** |
| *M.* cf. *tirolensis* | CZ 71E | Czechia, Svratka pond | 18.05.2020 | 49.7069431, 16.0270547 | 12.2 | 6.8 | 114 | **PV036689** |
| *M. tolerans* | CZ 229H | Czechia, Spálená Borkovna peat bog | 29.10.2023 | 48.9807556, 14.8003000 | 12 | 5.6 | 263 | **PV036690** |
| *M. tonsurata* | CZ 84A | Czechia, Odlehčovač canal | 09.10.2020 | 48.9696086, 14.8440406 | 10.5 | 7.3 | 140 | **PV036691** |
| *M. tonsurata* | CZ 32F | Czechia, alluvial pool 207 | 27.04.2020 | 48.86438, 14.89414 | 13.8 | 6.8 | 169 | **PV036692** |
| *M. torquata* | CZ 132E | Czechia, canal, Soutok | 08.11.2020 | 48.6628856, 16.9375286 | 8.7 | 7.5 | 908 | **PV036693** |
| *M. transsylvanica* | CZ 46F | Czechia, Velký Babín pond | 20.04.2020 | 49.5422786, 15.8974917 | 17.8 | 6.5 | 61 | **PV036694** |
| *M. trummensis* | CZ 192B | Czechia, alluvial pool T2 | 10.03.2022 | 48.8350986, 14.9270678 | 4.9 | 7.2 | 221 | **PV036695** |
| *M. tubulosa* | HU 04C | Hungary, Ilona-tó lake | 15.04.2022 | 47.7132772, 19.0403917 | 12.9 | 6.8 | 141 | **PV036696** |
| *S. americana* | S 81.C7 | Czechia, Záplavy | 19.03.2012 | 50.146470, 14.013906 | 5.2 | 7.2 | 670 | HG514166 |
| *S. asmundiae* | S 90.D11 | Finland, Rutajärvi lake | 05.05.2012 | 61.914025, 26.081592 | 6 | 5.4 | - | KP268729 |
| *S. bjoerkii* | S 90.B7 | Finland, Tehriselkä lake | 05.05.2012 | 61.756525, 26.485808 | 6 | 5.5 | - | **PV036697** |
| *S. conopea* | S 7.10 | Czechia, Babín pool | 2007 | 49.5421331, 15.8968439 | - | - | - | FM178506 |
| *S. curtispina* | CZ 08E | Czechia, alluvial pool Drnků | 12.03.2020 | 48.85259, 14.9087 | 10.5 | 8.2 | 228 | **PV036698** |
| *S. echinulate* | FI 12B | Finland, Rahelampi lake | 04.10.2021 | 62.8638111, 25.4435378 | 8.5 | 5.4 | 21 | **PV036699** |
| *S. glabra* | S 9.2 | Czechia, pool near Dyje river | 2007 | 48.676, 16.937 | - | - | - | FM178513 |
| *S. heteropora* | S 40.F11 | Czechia, Podhradská pool | 03.04.2011 | 50.461144, 14.911780 | 15.5 | 7.4 | 660 | HG514199 |
| *S. leptorrhabda* | S 89.C3 | Finland, unnamed pond | 05.05.2012 | 62.250297, 26.580000 | 5.6 | 7 | 40 | KP268752 |
| *S. macracantha* | S 90.B5 | Finland, Tehriselkä lake | 05.05.2012 | 61.756525, 26.485808 | 6 | 5.5 | - | KP268732 |
| *S. macropora* | S 71.B4 | Czechia, Podhradská pool | 04.03.2012 | 50.461145, 14.911741 | 5.5 | 6.5 | 230 | HG514228 |
| *S. multidentata* | S 90.C11 | Finland, Heinäjärvi lake | 05.05.2012 | 62.881234, 25.497420 | 7 | 5.9 | - | KP268755 |
| *S. petersenii* | S 7.7 | Czechia, Babín pool | 2007 | 49.542133, 15.896843 | - | - | - | FM178504 |
| *S. punctulosa* | W83 | Russia, flooded Bolshaya river | 28.06.2018 | 60.948180, 68.340860 | 9.8 | 7.7 | 69 | MW148515 |
| *S. rubra* | B57 | Czechia, Vizír pond | 9.10.2016 | 48.964652, 14.888559 | 7.9 | 6.6 | 89 | MK322808 |
| *S. sphagnicola* | K35 | Czechia, peat bog, Klečové louky | 04.10.2017 | 50.837274, 15.246051 | 18.4 | 3.9 | 51 | OP737841 |
| *S. spinosa* | S 88.F9 | Austria, Piburger See lake | 22.04.2012 | 47.195209, 10.886235 | - | 7.5 | 71 | **PV036700** |
| *S. splendida* | S 90.E4 | Finland, Rutajärvi lake | 05.05.2012 | 61.914025, 26.081592 | 6 | 5.4 | - | KP268761 |
| *S. truttae* | S 31.4 | Czechia, pond near Mezní Louka | 2007 | 50.877221, 14.319615 | - | - | - | FR819749 |
| *S. uvella* | S 61.M12 | Denmark, Bornholm, pool near Hundsemyre | 17.05.2011 | 55.030441, 15.105867 | 14.7 | 7.3 | 671 | **PV036701** |

**Table S4** The absolute numbers and relative abundances of Synurales species and species-level lineages, along with their functional traits. The genera are color-coded for clarity.

*Provided as a separate file.*

**Table S5** Average functional traits across the entire community of *Mallomonas*, *Synura*, and zooplankton species over the study period. Single color hues were used to emphasize temporal dynamics.

*Provided as a separate file.*
